# DyNAMiC: A prospective longitudinal study of dopamine and brain connectomes: A new window into cognitive aging

**DOI:** 10.1002/jnr.25039

**Published:** 2022-03-16

**Authors:** Kristin Nordin, Tetiana Gorbach, Robin Pedersen, Vania Panes Lundmark, Jarkko Johansson, Micael Andersson, Charlotte McNulty, Katrine Riklund, Anders Wåhlin, Goran Papenberg, Grégoria Kalpouzos, Lars Bäckman, Alireza Salami

**Affiliations:** ^1^ Umeå Center for Functional Brain Imaging (UFBI) Umeå University Umeå Sweden; ^2^ Department of Integrative Medical Biology Umeå University Umeå Sweden; ^3^ Wallenberg Centre for Molecular Medicine Umeå University Umeå Sweden; ^4^ Umeå School of Business, Economics and Statistics Umeå University Umeå Sweden; ^5^ Department of Radiation Sciences Umeå University Umeå Sweden; ^6^ Aging Research Center Karolinska Institutet & Stockholm University Stockholm Sweden; ^7^ Present address: Aging Research Center Karolinska Institutet & Stockholm University Stockholm 11330 Sweden

**Keywords:** aging, cognition, connectome, dopamine, lifespan, PET

## Abstract

Concomitant exploration of structural, functional, and neurochemical brain mechanisms underlying age‐related cognitive decline is crucial in promoting healthy aging. Here, we present the DopamiNe, Age, connectoMe, and Cognition (DyNAMiC) project, a multimodal, prospective 5‐year longitudinal study spanning the adult human lifespan. DyNAMiC examines age‐related changes in the brain’s structural and functional connectome in relation to changes in dopamine D1 receptor availability (D1DR), and their associations to cognitive decline. Critically, due to the complete lack of longitudinal D1DR data, the true trajectory of one of the most age‐sensitive dopamine systems remains unknown. The first DyNAMiC wave included 180 healthy participants (20–80 years). Brain imaging included magnetic resonance imaging assessing brain structure (white matter, gray matter, iron), perfusion, and function (during rest and task), and positron emission tomography (PET) with the [^11^C]SCH23390 radioligand. A subsample (*n* = 20, >65 years) was additionally scanned with [^11^C]raclopride PET measuring D2DR. Age‐related variation was evident for multiple modalities, such as D1DR; D2DR, and performance across the domains of episodic memory, working memory, and perceptual speed. Initial analyses demonstrated an inverted u‐shaped association between D1DR and resting‐state functional connectivity across cortical network nodes, such that regions with intermediate D1DR levels showed the highest levels of nodal strength. Evident within each age group, this is the first observation of such an association across the adult lifespan, suggesting that emergent functional architecture depends on underlying D1DR systems. Taken together, DyNAMiC is the largest D1DR study worldwide, and will enable a comprehensive examination of brain mechanisms underlying age‐related cognitive decline.

## Graphical abstract

This study presents the DopamiNe, Age, connectoMe, and Cognition (DyNAMiC) project, a multimodal, prospective 5‐year longitudinal study of the adult human lifespan. DyNAMiC examines age‐related differences in the brain’s structural and functional connectomes in relation to the dopaminergic (DA) D1 receptor system, and their associations to cognition in aging. Initial findings demonstrate an inverted u‐shaped effect of DA D1 receptor availability on functional connectivity across cortical network nodes.

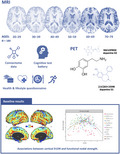

**Significance**

Simultaneous assessment of structural, functional, and neurochemical brain mechanisms underlying age‐related cognitive decline is crucial in promoting healthy aging. Longitudinal multimodal data are, however, currently lacking. We present the DyNAMiC project, which will ultimately examine changes in cognition, dopamine (DA), and the brain’s structural and functional connectome across the adult lifespan. DyNAMiC constitutes the largest DA D1 receptor study worldwide and also includes DA D2 assessment for a subsample of participants. Data from the first wave of DyNAMiC provide unique opportunities to examine contributions of different brain measures to individual differences in cognition, and to identify associations between functional and molecular brain systems as potential mechanisms of cognitive decline.

## 1 INTRODUCTION

The world’s elderly population is rapidly increasing and the number of individuals with age‐related cognitive impairments is expected to double over the next 50 years (United Nations, World Population Aging Report, 2015; 2019). Current knowledge of the brain mechanisms underlying age‐related cognitive decline is, however, insufficient to inform effective interventions promoting healthy aging. This is largely due to a scarcity of longitudinal multimodal data, hindering a comprehensive understanding of age‐related brain changes and their role in cognitive decline. In this paper, we describe the DopamiNe, Age, connectoMe, and Cognition (DyNAMiC) study, designed to examine changes in dopamine (DA) functions and in the brain’s structural and functional connectome across the adult lifespan, and the extent to which such changes impact cognitive decline in aging.

The human connectome describes elements and connections forming the human brain (Kelly et al., 2012; Petersen & Sporns, 2015; Sporns, 2013, 2014; Sporns et al., 2005). The structural connectome consists of white matter (WM) tracts interconnecting brain regions and represents the brain’s structural architecture, whereas the functional connectome, defined as synchronized activity across distal parts of the brain (i.e., functional connectivity; FC), reflects the functional architecture of complex neural systems. Spontaneous activity within the functional connectome at rest consumes the majority of the brain’s energy (Shulman et al., 2004), acts as a fingerprint (Finn et al., 2015), shows good test–retest reliability (Zuo & Xing, 2014), and persists during sleep and anesthesia (Vincent et al., 2007), suggesting that it is a rather stable trait of the brain. Numerous studies indicate correspondence between structural and functional connectomes (Damoiseaux & Greicius, 2009; Greicius et al., 2009; Hermundstad et al., 2013; Honey et al., 2009; Osmanlıoğlu et al., 2019; van den Heuvel et al., 2009), with structure–function associations varying across cortical regions (Baum et al., 2020; Vázquez‐Rodríguez et al., 2019). On this view, age‐related alterations in the functional connectome partly reflect changes in the structural connectome known to occur in aging (Betzel et al., 2014; Damoiseaux, 2017; Damoiseaux et al., 2009; Madden et al., 2009, 2020; Salami et al., 2012). Granted that cross‐sectional findings suggest variation in the relationship between structural and functional connectomes across the lifespan (Betzel et al., 2014), longitudinal observations are few and inconclusive (Fjell et al., 2016; Pedersen et al., 2021).

Regarding the functional connectome in aging, a recurrent cross‐sectional observation is decreased within‐network FC and increased between‐network FC in older adults (for reviews see Damoiseaux, 2017; Zuo et al., 2017), possibly reflecting neural dedifferentiation (i.e., reduced functional network specialization) in old age (Chan et al., 2014; Geerligs et al., 2014). Although changes in within‐network FC of large‐scale networks, such as the default mode network (DMN; Buckner et al., 2008; Raichle et al., 2001), have been reported in longitudinal studies (Salami et al., 2016), evidence for increased between‐network FC and changes in the overall functional architecture of the brain are limited (Ng et al., 2016; Pedersen et al., 2021). Different directions of age effects on within‐ and between‐network FC have further been observed comparing cross‐sectional and longitudinal estimates (Fjell et al., 2015). Relatedly, cross‐sectional studies report the functional connectome as predictive of cognition (Andrews‐Hanna et al., 2007; Damoiseaux et al., 2008; Wang et al., 2010), whereas longitudinal evidence of a link between age‐related cognitive decline and disruption in the functional connectome is limited (but see Malagurski et al., 2020; Pedersen et al., 2021).

It is possible that the integrity of the functional connectome and its link to cognition in aging is dependent on the integrity of the dopaminergic (DA) system, given that DA neurotransmission plays a key role in cognition through its modulation of synaptic activity enhancing specificity in the neuronal signal (El‐Ghundi et al., 2007; Seamans & Yang, 2004). Based on meta‐analyses, the two main postsynaptic DA receptor families, the DA D1 (D1DR) and D2 (D2DR) receptor families, show linear deterioration from early to late adulthood (Karrer et al., 2017; but see Seaman et al., 2019). Critically, cross‐sectional estimates of age‐related alterations of D1DR are most often limited to extreme age groups, with no study covering the adult lifespan (but see Rinne et al., 1990), and a balanced number of individuals per decade. Furthermore, no longitudinal data currently exist for D1DR, reported as the most age‐sensitive dopaminergic marker (Karrer et al., 2017). Thus, it remains unclear whether cross‐sectional estimates represent true rate and shape of D1DR decline across the lifespan.

In young adults, D1DR is associated with blood oxygenation level‐dependent (BOLD) brain activation (Turner et al., 2020) and to aspects of the functional connectome (Rieckmann et al., 2011; Roffman et al., 2016). Yet, to what extent the spatial configuration of the brain’s functional architecture (e.g., variation in nodal FC across regions) depends on underlying DA receptor distributions (Shine et al., 2019), and in what manner the relation between D1DR and the functional connectome changes across the lifespan remains unknown. In addition to addressing these questions, an important contribution of DyNAMiC will be to provide longitudinal estimates of D1DR (and the structural and functional connectome), enabling assessments of unique and shared mechanisms contributing to cognitive decline in aging. Concentrations of D1DR and D2DR vary across the brain (Ito et al., 2008), and DA is distributed through various distinct pathways (Haber, 2014). Accordingly, studies suggest that D1DR and D2DR may differentially contribute to prefrontal‐based processes of working memory and limbic‐based processes of episodic memory (Liggins, 2009; Nyberg et al., 2016; Takahashi et al., 2007, 2008). Thus, it is possible that D1DR and D2DR differentially contribute to specific aspects of the functional connectome, shaping their roles in age‐related cognitive decline.

Finally, brain and cognitive functions are characterized by a high degree of inter‐individual heterogeneity (Nyberg et al., 2012, 2020), likely reflecting both genetic and environmental factors. Various genetic and lifestyle factors (e.g., intellectual and physical activities), as well as vascular risk factors, all contribute to individual differences in cognitive aging (Dahle et al., 2009; Köhncke et al., 2018; Mintzer et al., 2019; Papenberg et al., 2015). For instance, physical activity measured over a decade may modify decline in some brain‐driven measures, including parts of the functional connectome (Boraxbekk et al., 2016).

DyNAMiC is a prospective 5‐year longitudinal study in collaboration between the Umeå Center for Functional Brain Imaging (UFBI) at Umeå University and the Aging Research Center (ARC) at Karolinska Institutet/Stockholm University, Sweden. The principal aims of DyNAMiC are to (1) determine the rates and trajectories of age‐related changes in different brain measures across the adult lifespan, focusing on the D1DR system and the brain connectome and; (2) delineate shared and unique contributions of changes in these brain measures to changes in various cognitive domains; and (3) identify factors contributing to altered brain integrity, such as genetic polymorphisms, health, and lifestyle factors (e.g., blood pressure, diet, and physical activity).

The specific aims of the present paper were, first, to provide a comprehensive overview of DyNAMiC and the baseline wave of data collection which included: (a) magnetic resonance imaging (MRI) for anatomical and functional brain measures; (b) PET scanning to assess postsynaptic DA systems with the D1DR radioligand [^11^C]SCH23390, making DyNAMiC the largest D1 study to date, and for a subset of participants an additional PET scan with the D2DR radioligand [^11^C]raclopride; (c) a cognitive test battery taxing age‐sensitive functions (e.g., episodic memory, working memory, and perceptual speed); (d) blood sampling for genetic factors related to DA functions and cognition; (e) evaluation of lifestyle factors such as medication, diet, and physical exercise. Our second aim was to present preliminary findings on global associations between the functional connectome and the DA D1 receptor system across the adult lifespan, using measures of resting‐state FC and D1DR from nodes of large‐scale cortical networks.

## 2 MATERIALS, METHODS, AND THE DyNAMiC DATABASE

### 2.1 Ethics statement

The DyNAMiC study was approved by the Regional Ethical board and the local Radiation Safety Committee in Umeå, Sweden. All participants provided written informed consent prior to testing, and all provided written consent for storage of blood samples at the Department of Biobank Research at the University Hospital of Umeå. For the PET scanning, ethical approval was granted for scanning the full sample (*n* = 180) with the DA D1 receptor radioligand [^11^C]SCH23390 (SCH‐PET), whereas only a subsample of 20 older individuals (age > 65 years) with both the DA D1 receptor radioligand [^11^C]SCH23390 (SCH‐PET) and the DA D2 receptor radioligand [^11^C]raclopride (RAC‐PET). The second PET assessment, carried out as an extension of the D1 scanning protocol, was restricted to a subsample of >65 years due to guidelines regarding radioactivity exposure along with well‐documented literature about onset of cognitive decline around this age.

### 2.2 Recruitment procedure

Participants (*n* = 180) were recruited from six decades from the age 20 to 80 years across the adult lifespan. Recruitment was ongoing throughout the first wave of data collection, between 2017 and 2020, with individuals included at baseline born between 1937 and 2000. Efforts were made to include approximately the same number of individuals from each decade of interest, and to achieve even distributions of age and sex (Table S1). Invitation letters were sent out to a sample randomly drawn (within each decade) from the population registry of Umeå, Sweden. The expected number of returnees for time point 2 was 120, based on previous longitudinal neuroimaging studies conducted in Umeå (COBRA: Nevalainen et al., 2015; and Betula: Nilsson et al., 1997, 2004), with attrition rates of ~30% between baseline and time point 2. Further information about the recruitment procedure is provided in the Supporting Information.

A set of exclusion criteria was implemented during recruitment to create a sample of healthy participants without conditions and medical treatment potentially affecting brain functioning and cognition. Respondents were excluded if they met one or more of the following criteria: brain injury or neurological disorder, dementia, neurodevelopmental disorder, psychiatric diagnosis, psychopharmacological treatment, history of severe head trauma, substance abuse or dependence, and illicit drug use. Individuals with other chronic or serious medical conditions (e.g., cancer, diabetes, and Parkinson’s disease) were also excluded.

Additionally, respondents had to meet the prerequisites for the study procedure in order to be included. This involved being able to undergo a 90‐min MRI scan, being able to see and hear adequately inside and outside the scanner environment, and being a native Swedish speaker. Thus, individuals having any non‐MRI safe metal implant or residue (e.g., pacemaker, medicine pump, neural stimulator, arterial clips, prostheses, splinters, and welding sparks) or MRI‐safe metal implant that might diminish image quality (e.g., titanium screw or permanent braces in the upper jaw) were excluded. Radiation safety was also taken into account before inclusion. Individuals having previously participated in a research project involving a PET scan, or who had recently undergone any other procedure involving the injection of a radioactively marked substance, were excluded. Pregnant women were also excluded, and breastfeeding women had to follow strict instructions (including no breastfeeding for at least 6 hr after the PET scan), in order to participate.

### 2.3 Participants

The recruitment process resulted in a final sample of 180 participants (90 men and 90 women, 20.5–78.7 years, *M* = 49.8 ± 17.4). Demographic information is presented in Table 1. Three participants dropped out during data collection. Thus, DyNAMiC includes 177 participants with baseline data from both the MRI and PET sessions. All participants underwent the Mini Mental State Examination (MMSE, Folstein et al., 1975), scoring ≥26.

**TABLE 1:** Sample characteristics

Variable	Value	Total sample		Age 20–29		Age 30–39		Age 40–49		Age 50–59		Age 60–69		Age 70–79	
		*N*	%	*N*	%	*N*	%	*N*	%	*N*	%	*N*	%	*N*	%
Sex	Female	90	50.0	14	48.3	16	53.3	14	48.3	14	48.3	15	50.0	17	51.5
	Male	90	50.0	15	51.7	14	46.7	15	51.7	15	51.7	15	50.0	16	48.5
	Total	180		29		30		29		29		30		33	
Age	Mean ± *SD*	49.8 ± 17.4		24.3 ± 2.8		34.1 ± 2.8		44.8 ± 2.6		53.9 ± 3.1		64.5 ± 2.7		74.0 ± 2.4	
MMSE	Mean ± *SD*	28.8 ± 1.2		29.6 ± 0.9		28.7 ± 1.2		29 ± 1.1		28.8 ± 1		28.4 ± 1.1		28.5 ± 1.3	
Education, level	Elementary (<10 years)	9	5.1					1	3.4			2	6.7	6	18.8
	High school (10–13 years)	60	33.7	12	42.9	7	23.3	7	24.1	12	41.4	13	43.3	9	28.1
	University (>13 years)	104	58.4	13	46.4	22	73.3	21	72.4	17	58.6	14	46.7	17	53.1
	NA	5	2.8	3	10.7	1	3.3					1	3.3		
	Total	178		28		30		29		29		30		32	
Education, years	Mean ± *SD*	14.9 ± 3.6		14.4 ± 2.2		16.1 ± 2.6		15.7 ± 3.4		15.2 ± 3.5		14.6 ± 4.1		13.4 ± 4.6	
Employment	Employed, full time	103	57.9	20	71.4	23	76.7	24	82.8	28	96.6	8	26.7		
	Employed, part time	20	11.2	3	10.7	5	16.7	4	13.8	1	3.4	5	16.7	2	6.2
	Retired	47	26.4									17	56.7	30	93.8
	Unemployed	7	3.9	4	14.3	2	6.7	1	3.4						
	NA	1	0.6	1	3.6										
	Total	178		28		30		29		29		30		32	
Accommodation	Cooperative apartment	42	23.6	8	28.6	9	30.0	5	17.2	7	24.1	4	13.3	9	28.1
	House	84	47.2	2	7.1	9	30.0	18	62.1	19	65.5	19	63.3	17	53.1
	Rental apartment	52	29.2	18	64.3	12	40.0	6	20.7	3	10.3	7	23.3	6	18.8
	Total	178		28		30		29		29		30		32	
Social status	Cohabitant	47	26.4	12	42.9	14	46.7	5	17.2	6	20.7	7	23.3	3	9.4
	Divorced	8	4.5					1	3.4	2	6.9	1	3.3	4	12.5
	Married	85	47.8			11	36.7	18	62.1	17	58.6	18	60.0	21	65.6
	Single	34	19.1	16	57.1	5	16.7	5	17.2	4	13.8	4	13.3		
	Widow/widower	4	2.2											4	12.5
	Total	178		28		30		29		29		30		32	
Children	0	50	28.1	27	96.4	10	33.3	5	17.2	1	3.4	6	20.0	1	3.1
	1–3	118	66.3	1	3.6	20	66.7	20	69.0	25	86.2	22	73.3	30	93.8
	>3	10	5.6					4	13.8	3	10.3	2	6.7	1	3.1
	Total	178		28		30		29		29		30		32	
	Mean ± *SD*	1 ± 1.9		0.1 ± 0.4		1.2 ± 1		1.9 ± 1.4		2.3 ± 0.9		1.8 ± 1.2		1.9 ± 1	
Grandchildren	0	123	69.1	28	100.0	28	93.3	28	96.6	20	69.0	12	40.0	7	21.9
	1–4	40	22.5					1	3.4	9	31.0	15	50.0	15	46.9
	>4	13	7.3									3	10.0	10	31.2
	NA	2	1.1			2	6.7								
	Total	178		28		30		29		29		30		32	
	Mean ± *SD*	1 ± 1.9		0 ± 0		0 ± 0		0.1 ± 0.4		0.6 ± 1		1.9 ± 2		3.2 ± 2.7	

*Note:* Age, sex, education, and socioeconomic factors across decades (means ± standard deviation; frequencies).

### 2.4 Study design

**FIGURE 1:**
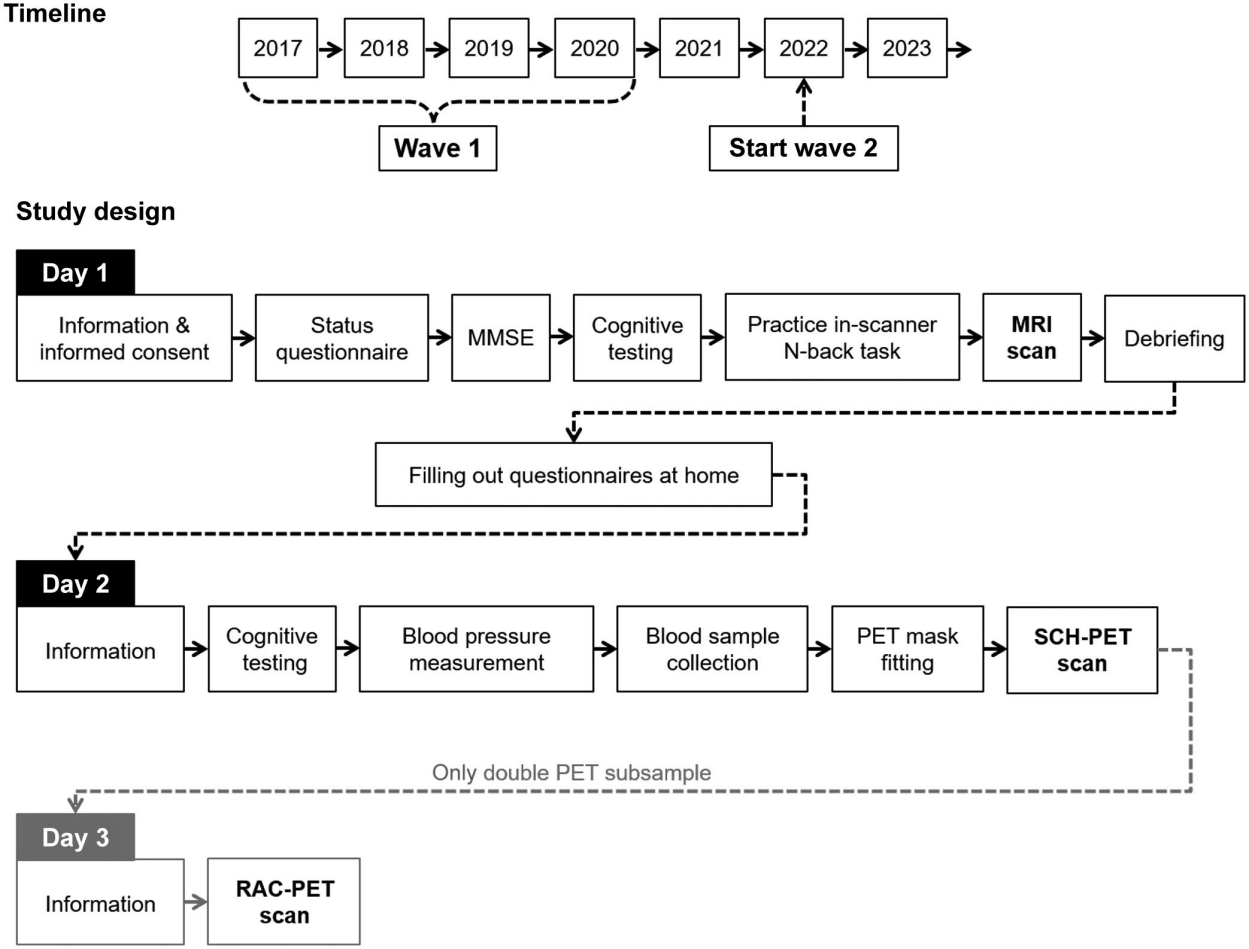
Overview of the DyNAMiC study timeline and design

DyNAMiC includes two time points of data collection. Data collection for the first time point was carried out in 2017–2020 at Umeå University Hospital. This will remain the same for the second time point, planned to follow 5 years from baseline, starting in 2022 (see Figure 1). Participants will be scheduled for testing in an order corresponding to their participation at baseline. All included procedures and testing will be the same for time point 2 as for the baseline measurement. At each wave, testing is distributed over 2 or 3 separate days, including one MRI session and one PET session (SCH‐PET to assess D1DR) for the full sample, as well as a second PET session (RAC‐PET for assessment of D2DR) for a subsample of participants (*n* = 20; >65 years of age). Testing procedures for the different sessions are outlined in Figure 1.

The first session included the MMSE, testing of specific cognitive functions, and MRI assessment, and lasted approximately 3 hr 45 min. Participants provided written informed consent at the beginning of the session. Before MRI scanning, which lasted for 90 min, they responded to a short status questionnaire assessing their current alertness with questions of sleep and caffeine intake, and practiced the in‐scanner working memory *n*‐back task. Participants also received a comprehensive lifestyle questionnaire to fill out at home. The second session included cognitive testing, blood pressure measurement, blood sampling, and a 60 min SCH‐PET scan following the individual fitting of a thermoplastic mask for in‐scanner head stabilization. This session lasted approximately 2 hr 30 min in total. For the subsample of older participants, a third session included a 60 min RAC‐PET scan using the fitted mask from their first PET session.

For the larger group of participants, completing only the SCH‐PET session, data collection proceeded as planned for 83% (*n* = 130), with an average of 2 days between MRI and PET sessions (ranging from 1 to 10 days). For the remaining 17%, data collection was delayed, resulting in an average of 55 days between sessions (ranging between 18 and 141 days). For the majority of the smaller, double‐PET subsample (80%, *n* = 16), data collection was completed with an average of 10 days in between the first and last session (ranging from 7 to 11 days). PET sessions were delayed for the remaining four participants, with the number of days between the first and last session ranging from 35 to 49. The most common reason for delay between sessions was PET tracer production failure.

### 2.5 Cognitive measures

**FIGURE 2:**
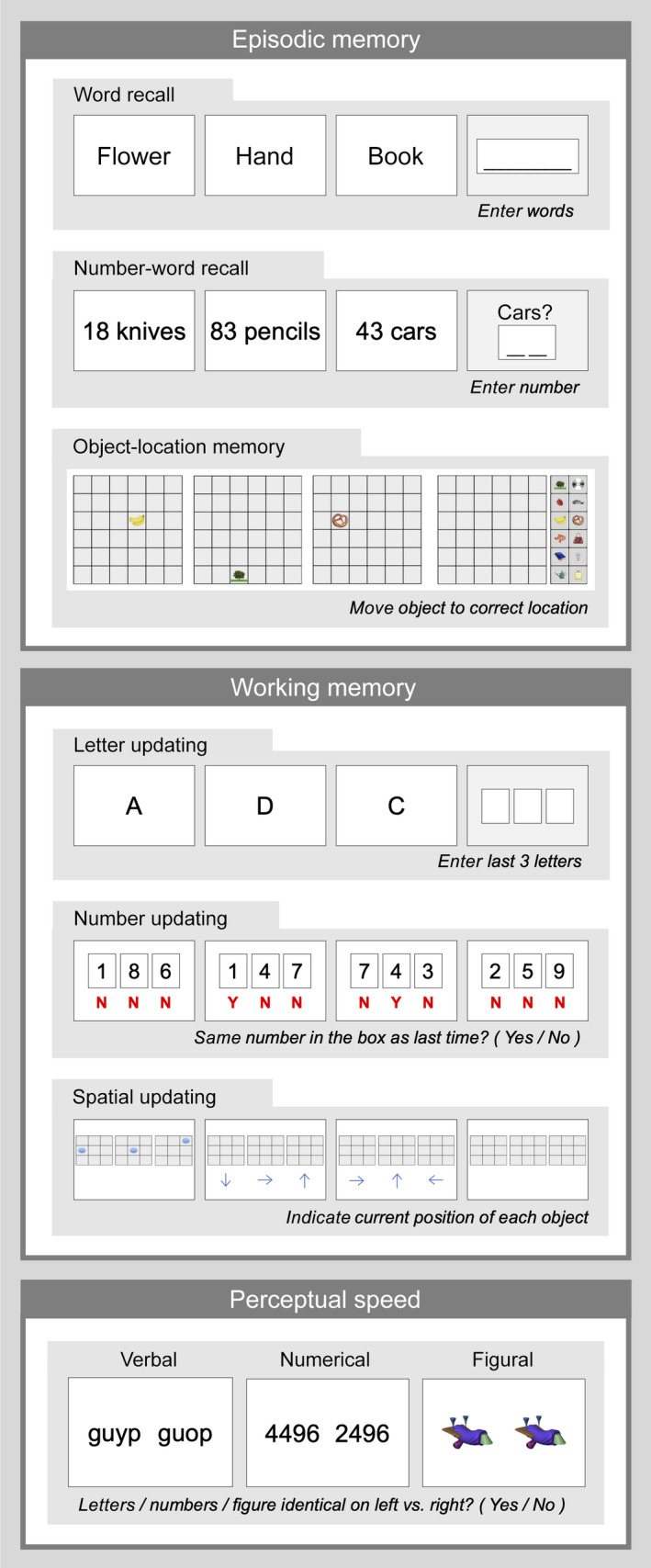
Overview of the main cognitive tests included in DyNAMiC

A battery of cognitive tests assessed episodic memory, working memory, and perceptual speed. This battery was originally developed for the COGITO study (Schmiedek, Bauer, et al., 2010; Schmiedek, Lövdén, et al., 2010), and later adapted for the Umeå‐based COBRA study including Swedish participants (Nevalainen et al., 2015). Each of the three cognitive domains was assessed using three separate tasks containing letter‐, number‐, and figure‐based material, respectively (Figure 2). Tasks were presented on a computer, in the same order across participants, which provided their responses by either typing in words or numbers; using the computer mouse; or pressing keys marked by different colors corresponding to specific response alternatives. Every task started with a written instruction, after which one or several practice runs were completed (varying across tasks). Testing then followed in several runs, resulting in the overall performance (e.g., accuracy, response times, or frequencies) being a composite of performance across the separate test runs. Testing was conducted across two sessions prior to MRI and PET scanning (Figure 1). A test leader was present throughout both sessions. This section provides a description of the episodic memory, working memory, and perceptual speed tests, as well as included tests of semantic knowledge, implicit learning, and motor speed.

Reliability measures of cognitive tests including two trials were estimated using the Spearman–Brown coefficient, which might be less biased than Cronbach’s alpha (Eisinga et al., 2013). Nevertheless, differences between Spearman–Brown coefficients and Cronbach’s alpha for our measures were small (<0.01). Cronbach’s alpha was used as the measure of reliability for cognitive tests including more than two trials.

#### 2.5.1 Episodic memory

Tests of episodic memory included word recall, number‐word recall, and object‐location recall (Figure 2). In word recall, participants were presented with 16 words that appeared one by one on the computer screen. Words were concrete Swedish nouns (e.g., flower) and no two words shared the same first three letters. During the first phase, participants encoded each word for 6 s, with an inter‐stimulus interval (ISI) of 1 s. Following the presentation of all words in the series, participants used the keyboard to type in as many of the presented words that could recall, in any order. Performance was defined as the number of correctly recalled words. Two trials of this test were completed after an initial practice trial, yielding the maximum score of 32. The reliability of this measure across the two trials was 0.86 (Spearman–Brown coefficient, based on *n* = 180 subjects).

In the number‐word recall test, participants were required to memorize pairs of two‐digit numbers and concrete plural nouns (e.g., 46 dogs). Ten number‐word pairs were presented consecutively, each displayed for 6 s, with an ISI of 1 s. Retrieval immediately followed, in which every word was consecutively presented again, but in a different order than during encoding. For each word, participants had to recall the associated two‐digit number, and type the answer using the keyboard. A response was required for each word, meaning that participants had to provide a guessing‐based response even if they did not recall the correct number. Following an initial practice trial, this test was administered in two trials with a total maximum score of 20 correctly recalled numbers. This test showed a reliability of 0.76 (Spearman–Brown coefficient, *n* = 180) across trials.

In the object‐location memory task, participants encoded objects presented on different locations in a 6 × 6 square grid displayed on the computer screen. Each encoding trial involved 12 objects, one by one, in distinct locations within the grid. Each object‐position pairing was displayed for 8 s before disappearing, with an ISI of 1 s. Directly following encoding, all objects were simultaneously displayed next to the grid for participants to move them (in any order) to their correct location in the grid. If unable to recall an object’s correct position, participants had to guess and place the object at a location to the best of their ability. Two test trials of this task were administered after a practice trial, yielding a total maximum score of 24. The reliability of this measure was 0.69 (Spearman–Brown coefficient, *n* = 180).

#### 2.5.2 Working memory

Working memory was also tested using three tasks, letter updating, number updating, and spatial updating (Figure 2). These three tests were different from the working memory *n*‐back task performed by participants during fMRI scanning (described in Section 2.8.1.5.). During letter updating, participants were presented with a sequence of capital letters (A–D), consecutively on the computer screen, requiring them to update and to keep the three lastly presented letters in memory. The letters were presented for 1 s, with an ISI of 0.5 s. When prompted, which could be at any given moment, participants provided their response by typing in three letters using the keyboard. If they failed to remember the correct letter, they provided a guessing‐based answer. Four practice trials were completed by all participants, followed by 16 test trials consisting of either 7‐, 9‐, 11‐, or 13‐letter sequences. Across all 16 trials, the maximum number of correct answers were 48 (16 trials × 3 reported letters = 48). The estimated reliability of this measure was 0.783 (Cronbach’s alpha, *n* = 179).

The number‐updating task had a columnized numerical 3‐back design. Three boxes were present on the screen throughout the task, in which a single digit (1–9) was presented one at a time, from left to right during 1.5 s with an ISI of 0.5 s. During this ongoing sequence, participants had to judge whether the number currently presented in a specific box matched the last number presented in the same box (appearing three numbers before). For each presented number they responded yes/no by pressing one of two assigned keys (“yes” = right index finger; “no” = left index finger). Four test trials, each consisting of 30 numbers, followed after two practice trials. Performance was defined as the sum of correct responses across the four test trials, after discarding responses to the first three numbers in every trial (as these were not preceded by any numbers to be matched with). The maximum score was 108 (27 numbers × 4 trials). Reliability of this measure was estimated as 0.95 (Cronbach’s alpha, *n* = 179).

In the spatial‐updating task, three 3 × 3 square grids were presented next to each other on the computer screen. At the beginning of each trial, a blue circular object was, at the beginning of each trial, displayed at a random location within each grid. Following a presentation time of 4 s, the circular objects disappeared, leaving the grids empty. An arrow then appeared below each grid, indicating that the circular object in the corresponding grid was to be mentally moved one step in the direction of the arrow. The arrows appeared stepwise from the leftmost grid to the rightmost grid, each presented for 2.5 s (ISI = 0.5 s). The exercise of mentally moving the circular object was repeated one more time for each grid, prompted by three new arrows, resulting in the object having moved two steps from its original location at the end of each trial. Using the computer mouse, participants then indicated which square the circular object in each grid had ended up in. If unsure, they provided guesses. The test was performed across 10 test trials, preceded by five practice trials. Performance was calculated as the sum of correct location indications across trials, with a maximum score of 30. The reliability for this test was 0.84 (Cronbach’s alpha, *n* = 178).

#### 2.5.3 Perceptual speed

Three tasks assessed perceptual speed: letter comparison, number comparison, and figure comparison (Figure 2). Although the type of material varied across the three tasks, they all had a similar design. Participants were required to judge whether two items presented next to each other on the screen were identical or not, and provided yes/no responses by pressing assigned keys on the keyboard. The instructions were to respond as correctly and fast as possible.

In letter comparison, items consisted of two strings of four letters (a–z), constructed as to not constitute real words. Two strings were identical if they included the same letters in the same sequence, and non‐identical when one letter differed between them. An item pair was presented until a response was provided, or for a maximum of 5 s (timeout). The ISI was 0.5 s. Each test trial consisted of 40 item pairs, half of which were identical and the other half were different. Testing took place over two test trials, preceded by one initial practice trial. Performance was calculated by dividing the number of correct responses by the total response time in milliseconds (i.e., the response time for both correct and incorrect responses), and then multiplying by 60,000 to create a score of correct responses per minute while imposing a penalty on incorrect responses. Scores were then summed across the two test trials. The summed performance score had a reliability of 0.975 (Spearman–Brown coefficient, *n* = 180).

In the number comparison test, item pairs consisted of two strings of four digits (1–9). The design and procedure were in all other aspects equal to the letter comparison test. Performance was calculated in the same way (i.e., number of correct responses per minute), and summed across two test trials. The reliability of this score was 0.968 (Spearman–Brown coefficient, *n* = 180).

The figure‐comparison task was similar to the letter and number comparison tasks in its design and procedure, although including item pairs consisting of figures (“fribbles,” not representing any real objects; provided by Michael J Tarr, Brown University, Providence, RI, USA, http://www.tarrlab.org). These figures were built of multiple components and considered identical when matching in all of their components, and not matching when identical except for one component. Performance was calculated in the same way as for the other comparison tasks, and the reliability of this measure across two test trials was 0.95 (Spearman–Brown coefficient, *n* = 180).

#### 2.5.4 Semantic knowledge

Semantic knowledge was tested through a vocabulary test, using the Synonyms test from the Dureman‐Sälde battery (SRB: Dureman, 1960). Participants were presented with 30 Swedish words, each displayed along with five additional words, one of which was a synonym to the target word. Participants had to select which word they believed to be the synonym to the target word, and provided their responses by clicking a corresponding check box. This task was self‐paced, and apart from one example word with synonyms in the beginning, no practice run was administered. Each correctly identified synonym gave participants 1 point, making the total maximum score 30.

#### 2.5.5 Implicit learning

Implicit learning was operationalized as sequence learning and tested using the serial‐reaction time test (SRTT: Nissen & Bullemer, 1987; Rieckmann et al., 2010; Seger, 1994). This task presents participants with sequences of events that they have to respond to, and measures the difference in reaction time between repeated and new sequences, considered to reflect implicit sequence learning. In this task, four squares were presented next to each other in a row on the computer screen. The squares were grouped together in two pairs (one more to the left and the other more to the right of the center of the screen). This way, there was a spatial correspondence between the squares and the response buttons on the keyboard: The two squares to the left corresponded to participants' left middle and index fingers, whereas the two squares to the right corresponded to participants' right middle and index fingers. The instructions were to press the correct key, as fast as possible, every time the corresponding square on the screen changed color from white to dark. Squares were dark for 750 ms, during which participants had to provide their response. The ISI was either a maximum of 750 ms if no response was provided (timeout), or 250 ms in cases where a key had been pressed. Each of six test trials included 48 items (color‐change events). Blocks 1–4 and 6 consisted of identical second‐order 12‐item sequences repeated four times, and block 5 consisted of four repetitions of new second‐order 12‐item sequences. The task was presented as a motor speed task, and participants were not informed about the presence of these sequences. Implicit sequence learning was measured as the difference in response time between repeated and new sequences (block 5 − [block 4 + block 6]/2). Two practice trials were initially performed. These consisted of two blocks with 24 items each (with sequences non‐overlapping with test trials).

#### 2.5.6 Motor speed

Motor speed was assessed using a finger‐tapping test measuring participants' maximum finger‐tapping frequency. For each hand, one practice trial and one test trial was administered. The instructions were to place the index finger of the hand being tested on a given computer key and, when cued, tap as fast as possible for the duration of the trial which was 25 s.

### 2.6 Blood sampling

All participants provided a blood sample at the second testing session. These samples are stored at the Department of Biobank Research at Norrlands University Hospital in Umeå, Sweden. Samples were collected for analyses of genetic and metabolomic factors potentially contributing to individual differences in cognition, brain measures, and age‐related changes therein. Fasting was not required. A total of 40 ml blood was obtained, in equal amounts in four separate tubes, using a 1.3 mm diameter cannula. The time of blood sampling was registered and the samples were transported to the biobank for storage (at −80°C), maximum 2 hr after collection. Extraction of DNA will be performed for genotyping of single nucleotide polymorphisms of genes coding for DA genes (e.g., DA D1/2, COMT) as well as their methylation profiles to assess epigenetic factors. Other genetic polymorphisms related to brain integrity and cognition will also be assessed (e.g., APOE).

### 2.7 Questionnaires: Demographics, health, and lifestyle

Participants filled out several questionnaires (all used in their Swedish version), covering demographic variables, health, and lifestyle factors. Questionnaire data are available for 178 participants. Demographic and socioeconomic sections included factors such as education, marital status, number of children, and type of accommodation (Table 1). Health and lifestyle factors included questions on diet, dietary supplements, and use of medications (Table 2). Additionally, participants provided a detailed list of prescribed medications currently used, and responded to questions about their dental health. A screening instrument for hazardous alcohol use, abuse, and dependence was also included (Alcohol Use Disorders Identification Test; Babor et al., 2001; Berman et al., 2012). Frequency and intensity of activity within three main domains were assessed (Nevalainen et al., 2015; Table S3): social activities, cognitive activities, and physical activities.

**TABLE 2:** Objective and self‐reported health measures

Variable	Total sample		Age 20–29		Age 30–39		Age 40–49		Age 50–59		Age 60–69		Age 70–79	
	*N*	%	*N*	%	*N*	%	*N*	%	*N*	%	*N*	%	*N*	%
BMI	25.3 ± 4.1		24.3 ± 3.7		25.1 ± 4.6		26.7 ± 3.7		24.8 ± 3.3		25.7 ± 4.9		25 ± 4.1	
Length	1.7 ± 0.1		1.8 ± 0.1		1.7 ± 0.1		1.7 ± 0.1		1.7 ± 0.1		1.7 ± 0.1		1.7 ± 0.1	
Weight	75.6 ± 15.4		75.2 ± 13.8		75.7 ± 19		80.2 ± 13.8		75.6 ± 14.5		75.1 ± 14.6		72.2 ± 16.4	
*Nicotine*														
No	141	79.2	19	67.9	23	76.7	24	82.8	24	82.8	24	80.0	27	84.4
Yes, smoke	10	5.6	4	14.3	1	3.3	0	0.0	0	0.0	1	3.3	4	12.5
Yes, snus	21	11.8	2	7.1	6	20.0	4	13.8	5	17.2	3	10.0	1	3.1
Yes, smoke and snus	4	2.2	2	7.1	0	0.0	1	3.4	0	0.0	1	3.3	0	0.0
NA	2	1.1	1	3.6	0	0.0	0	0.0	0	0.0	1	3.3	0	0.0
Total	178		28		30		29		29		30		32	
*Medication*														
Hypertension	28				1		4		1		7		15	
Asthma	14		4		2		5		2				1	
Hyperlipidemia	12								2		4		6	
Cardiovascular disease/risk factors	11										3		8	
Hypothyroidism	11								2		6		3	
Musculoskeletal pain	11				1		3		1		4		2	
Gastrointestinal disease	8		2		1		1				2		2	
Allergy	7		2		4				1					
Prophylaxis	7		1		1				1		1		3	
Contraception	5		1		3		1							
Osteoporosis	4										1		3	
Migraine	3								3					
Rheumatic diseases	3										1		2	
Sleep disorders	3		1								1		1	
Menopause symptoms	2										1		1	
Other^a^	18		2		1		2		2		4		7	

*Note:* Data are presented as frequencies and mean values (± standard deviation).

^a^ The category “other” includes, among others, the following conditions: benign prostatic hyperplasia, chronic obstructive pulmonary disease, depression and/or anxiety, inflammation, movement disorders, musculoskeletal pain or substance abuse/dependence.

Additional questionnaires assessed handedness (a Swedish adaptation of the Edinburgh Handedness Inventory; Oldfield, 1971), personality (Big Five Inventory; John & Srivastava, 1999; Zakrisson, 2010), subjective memory function (Prospective and Retrospective Memory Questionnaire; Crawford et al., 2003; Rönnlund et al., 2008), and mental health variables such as levels of perceived stress (10‐item Perceived Stress Scale; Cohen et al., 1983; Nordin & Nordin, 2013), sleep disturbances (Karolinska Sleep Questionnaire; Ingre et al., 2000), depression symptoms (Beck Depression Inventory; Beck et al., 1996), pathological worry (Penn State Worry Questionnaire, translated to Swedish by Breitholtz & Rondahl, 2004; Meyer et al., 1990), and dementia worry (in‐house Swedish translation of the Dementia Worry Scale; Suhr & Isgrigg, 2011).

### 2.8 Brain imaging

Structural, functional, and neurochemical brain measures were acquired using MRI and PET at Umeå Center for Functional Brain Imaging (UFBI) and Umeå University Hospital in Umeå, Sweden. This section includes the imaging parameters for all imaging modalities, whereas preprocessing procedures are described in a subsequent section for a selected number of measures reported in the present study (see Section 2.9).

#### 2.8.1 Magnetic resonance imaging (MRI)

MR data were obtained with a 3T Discovery MR 750 scanner (General Electric), equipped with a 32‐channel phased‐array head coil. Participants were provided information about the sequences (i.e., resting state, movie watching, and a working memory *n*‐back task) and scanning procedures prior to each session. They were also instructed to lie as still as possible throughout the examination and were fitted with earplugs and headphones to minimize scanner‐noise exposure. Head motion was additionally minimized using cushions placed inside the head coil. An MR‐compatible response box was provided for participants to perform the in‐scanner task (*n*‐back), using their right hand. Cardiac and respiratory recordings were acquired during each scan using photoplethysmography fitted to participants' left index finger, and a pneumatic belt placed around the subject’s abdomen. Recordings were sampled at 100 and 25 Hz for cardiac and respiratory traces, respectively, using an automatic start 30 s prior to each MRI sequence. Participants had access to an alarm button to alert medical staff and test leaders during image acquisition if needed. All participants had normal or corrected‐to‐normal vision using contact lenses or scanner‐compatible glasses following a visual acuity test prior to scanning.

##### Structural MRI

High‐resolution anatomical T1‐weighted images were collected using a 3D fast spoiled gradient‐echo sequence. Imaging parameters were as follows: 176 sagittal slices, thickness = 1 mm, repetition time (TR) = 8.2 ms, echo time (TE) = 3.2 ms, flip angle = 12°, and field of view (FOV) = 250 × 250 mm.

##### White matter microstructure and integrity

Diffusion weighted imaging (DWI) was conducted to assess WM integrity. Images were collected using a multiband echo planar imaging sequence, with 90 independent directions. The total slice number was 63, with TR = 3,000 ms, TE = 73.0 ms, flip angle = 90°, FOV = 256 × 256 mm, and *b* = 2,000 s/mm^2^. Multiband acceleration factor = 3, in‐plane acceleration factor = 2. Two sets of 10 *b* = 0 baseline images were collected with opposing polarities in the phase‐encoding direction. This allows for correction of spatial distortions due to susceptibility‐induced magnetic field inhomogeneities.

For assessment of WM hyperintensities, a fluid‐attenuated inversion recovery (FLAIR) sequence was acquired. A total of 48 slices were acquired with a slice thickness of 3 mm, TE = 120 ms, TR = 8,000 ms, TI = 2,250 ms, and FOV = 240 × 240 mm.

##### Cerebral perfusion

In order to assess cerebral perfusion, images were sampled using 3D pseudo‐continuous arterial spin labeling (3D pcASL) with background suppression and a spiral readout. The total scanning time was approximately 4.5 min, with a labeling time of 1.5 s, post‐labeling delay time of 2 s, FOV = 240 × 240 mm, slice thickness of 4 mm, and an acquisition resolution of 8 × 512 (8 arms with 512 data points) with the number of averages set at 3. This sequence provided whole‐brain perfusion in ml/100 g/min.

##### Brain iron measurements

To investigate brain iron accumulation, a 3D multi‐echo gradient‐recalled echo (meGRE) sequence was used with the following parameters: voxel size = 1 × 1 × 1.2 mm^3^, TR = 31 ms, FOV = 256 × 256 mm, no gap, 124 axial slices, and flip angle = 17°. The first TE was 1.78 ms, followed by seven additional TEs with 2.872 ms intervals.

##### Functional MRI

Whole‐brain functional images were acquired during three conditions: resting state, naturalistic viewing, and working memory. Data from the resting‐state condition are presented in this study, while descriptions of the other two conditions are included in the Supporting Information. Resting‐state functional images were sampled using a T2*‐weighted single‐shot echo‐planar imaging (EPI) sequence, with a total of 350 volumes collected over 12 min (which is shown to yield a decent identification accuracy for the functional connectome [Finn et al., 2015]). The functional sequence was sampled with 37 transaxial slices, slice thickness = 3.4 mm, 0.5 mm spacing, TR = 2,000 ms, TE = 30 ms, flip angle = 80°, and FOV = 250 × 250 mm. Ten dummy scans were collected at the start of the sequence.

Participants were instructed to stay awake, keep their eyes open, and focus on a white fixation cross presented on a black background displayed on a computer screen seen through a tilted mirror attached to the head coil. Arousal was not monitored, but following scanning, participants provided information about their ability to stay awake during the sequence.

#### 2.8.2 Positron emission tomography (PET)

PET was conducted in 3D mode with a Discovery PET/CT 690 (General Electric, WI, US) to assess whole‐brain D1DR (using [^11^C]SCH23390) and D2DR (using [^11^C]raclopride) at rest. Both ligands were produced at Umeå University Hospital. Three participants did not receive the [^11^C]SCH23390 scan because of technical and personal reasons, one participant aborted the [^11^C]SCH23390 examination after approximately 40 min due to discomfort caused by the headrest, and one participant’s [^11^C]SCH23390 injection did not enter arterial blood directly as deemed by markedly slower appearance of signal in the brain, leaving 175 participants with complete and coherent [^11^C]SCH23390 data. There were no reported deviations in [^11^C]raclopride examinations.

Each scanning session started with acquiring a low‐dose CT for attenuation correction with 10 mA, 120 kV, and 0.8 s rotation time. Participants were instructed to lay still and remain awake with eyes open, while external stimuli were kept at minimum during the PET examinations. To minimize head movement, a thermoplastic mask (Posicast®; CIVCO medical solutions; IA, US) attached to the bed surface during scanning, was individually fitted for each participant. Mask molding was done through first softening the mask by soaking in warm water (approximately 73°C), then placing it over and around the participant’s face. Finally, the mask was removed when dried and solidified in the preferred shape. For participants completing both PET sessions, masks created at the first session were reused at session 2.

##### [^11^C]SCH23390 PET

An intravenous bolus injection with target radioactivity of 350 MBq [^11^C]SCH23390 was administered at the start of a 60‐min dynamic PET scan, with 6 × 10 s, 6 × 20 s, 6 × 40 s, 9 × 60 s, and 22 × 120 s frames. The average radioactivity dose administered to participants was 337 ± 27 MBq (range 205–391 MBq).

##### [ ^11^C]raclopride PET

Following the 5‐min mark of scan onset, an intravenous bolus injection of [^11^C]raclopride, prepared to be 250 MBq at the time of injection, was given to participants. The average radioactivity dose received by participants was 275 ± 15 MBq (range 238–305 MBq). A 55‐min, 60‐frame, dynamic scan (10 × 30 s and 50 × 60 s) was then acquired directly following injection.

### 2.9 Processing of brain imaging data

The preprocessing and analysis of selected measures of MRI and PET data are described in this section. The measures include the anatomical T1‐weighted images, resting‐state fMRI data, the [^11^C]SCH23390 and [^11^C]raclopride PET data.

#### 2.9.1 Volumetric assessments

Anatomical T1‐weighted images were used to delineate subcortical structures with the Freesurfer 6.0 software (https://surfer.nmr.mgh.harvard.edu; Fischl et al., 2002). Striatal volumes were manually corrected using the Voxel Edit mode in Freeview when necessary. The number of voxels within delineated structures represented gray and white matter volumes. Raw volumes were corrected for estimated total intracranial volume (eTIV) prior to analyses, such that adjusted volume = raw volume − *b*(eTIV – mean eTIV), where *b* is the slope of regression of volume on eTIV (Buckner et al., 2004; Jack et al., 1989). Distributions of regional gray matter volumes are presented in the Figure S1.

#### 2.9.2 Functional MRI

Functional data from the resting‐state condition were preprocessed following steps described in prior work (Avelar‐Pereira et al., 2020; Gorbach et al., 2020; Pedersen et al., 2021; Salami et al., 2014). Functional images underwent slice‐timing and movement correction, followed by distortion correction using subject‐specific field maps. Three participants were excluded from the distortion correction procedure due to technical issues during field‐map acquisition. Structural and functional data were subsequently co‐registered and normalized using a study‐specific template by Diffeomorphic Anatomical Registration using Exponentiated Lie algebra (DARTEL: Ashburner, 2007). Four individuals were excluded in the template‐generation step due to non‐pathological anatomical deviations. Following DARTEL, subject‐specific flow fields were used to normalize images to MNI space, and images were subsequently smoothed with a 6‐mm Gaussian kernel.

Additional preprocessing steps were completed to reduce spurious variance from non‐neuronal sources: (i) demeaning and detrending each run, (ii) defining a multiple regression model including several nuisance regressors described below, (iii) finally, nuisance regression as setup in the previous step and temporal high‐pass frequency filtering (threshold of 0.009 Hz) were applied simultaneously to not re‐introduce nuisance signals (Hallquist et al., 2013). The nuisance regressors included mean cerebrospinal and white matter signal, Friston’s 24‐parameter motion model (six motion parameters, their squares and temporal derivatives; Friston et al., 1996), and a binary vector of motion‐contaminated volumes identified by the degree of frame wise displacement (FD). An FD metric that is independent of the definition of the center of rotation was used based on the transformation matrix instead of rotation directly (Jenkinson et al., 2002). Volumes with FD > 0.2 mm were flagged as motion contaminated. Finally, physiological nuisance regressors were included to control for spurious effects of respiration and heart rate using the Matlab PhysIO Toolbox v.5.0 (Kasper et al., 2017). A RETRICOR model (Glover et al., 2000; Hutton et al., 2011) was employed using Fourier expansions for the estimated phases of cardiac pulsation (up to third‐order harmonics), respiration (up to forth‐order harmonics), and first‐order cardio‐respiratory interactions.

#### 2.9.3 PET data

PET data were processed for two separate purposes. First, a characterization of striatal D1DR and D2DR, for which data processing followed the same steps for [^11^C]SCH23390 and [^11^C]raclopride images. Striatal regions were selected for baseline characterization of D1DR and D2DR in the DyNAMiC sample based on previous findings linking structural, functional, and DA receptor integrity of these regions to age‐sensitive cognitive domains (Bäckman et al., 2000, 2010, 2011; Nyberg, 2017). Furthermore, given their rich DA innervation, striatal regions serve as a good point of reference in terms of D1DR and D2DR estimates, as well as across the DyNAMiC and COBRA (Nevalainen et al., 2015) data sets enabling comparisons and pooling of PET data. The second purpose was for estimation of D1DR in cortical regions corresponding to nodes in known functional brain systems (Power et al., 2011). These estimates were later used in assessments of associations between D1DR and functional connectivity across cortical regions.

For both purposes, binding potential relative to non‐displaceable binding in a reference region (BP_ND_; Innis et al., 2007), was used as an estimate of receptor availability (i.e., D1DR; D2DR) in target regions, using the cerebellum as reference. PET data were corrected for head movement by using frame‐to‐frame image co‐registration, and co‐registered with T1‐weighted MRI data with re‐slicing to MR voxel size using Statistical Parametric Mapping (SPM12: Wellcome Trust Centre for Neuroimaging, http://www.fil.ion.ucl.ac.uk/spm/). For the striatal regions putamen and caudate nucleus, the simplified reference tissue model (SRTM) was used to model regional time‐activity course (TAC) data (Lammertsma & Hume, 1996). Regional TAC data were adjusted for partial volume effects (PVEs) by using the symmetric geometric transfer matrix (SGTM) method implemented in FreeSurfer (Greve et al., 2016), and an estimated point spread function of 2.5 mm full width at half maximum (FWHM).

Estimation of D1DR in cortical regions was based on voxel‐wise BP_ND_ maps, computed by using multilinear SRTM with fixed *k*_2_’ (MRTM2; Ichise et al., 2003). For each participant, average voxel‐wise estimates were extracted for 243 cortical regions (5‐mm‐radius spheres) defined in the Power atlas (Power et al., 2011). Voxel‐wise TAC were adjusted for PVE by using the Muller‐Gartner method implemented in FreeSurfer 6.0 (Greve et al., 2016), with PSF 2.5 mm FWHM, and spatially normalized to match the coordinates in Montreal Neurological Institute (MNI) template.

#### 2.9.4 Associations between cortical functional connectivity and D1DR

The second aim of the current study was to provide an initial characterization of associations between the functional connectome and D1DR. To that end, we present analyses and initial results from ongoing work in our group. To characterize the functional connectome, we employed a graph theoretical approach to quantify resting‐state functional connectivity in terms of ROI‐wise nodal strength of 243 cortical regions that have shown to be in good agreement with known functional brain systems (Power et al., 2011). A functional connectome was created for each participant following data preprocessing by extracting average resting‐state fMRI time series for each cortical region defined in the Power parcellation (defined as 5‐mm‐radius spheres). The extracted time series were correlated using Pearson’s correlations followed by Fisher’s r‐to‐z transformation. Using the Brain Connectivity Toolbox (https://sites.google.com/site/bctnet/), average nodal strength of positive edges (e.g., connections between nodes) was subsequently computed for each ROI, based on data for participants having completed both resting‐state fMRI and [^11^C]SCH23390 PET (*n* = 175). To avoid age‐related bias in the distribution of positive edges, only edges found to be positive in at least half of the sample were considered. Nodal D1DR was defined as the estimates of cortical D1DR extracted from each functional ROI during preprocessing, averaged across participants. The same connectivity and D1DR estimates were also computed within each decade, and for three larger groupings of young (20–39 years, *n* = 57), middle‐aged (40–59 years, *n* = 57), and older adults (60–79, *n* = 62). Stepwise linear and quadratic modeling was then conducted to investigate the link between D1DR and nodal strength.

### 2.10 Statistical power

Because DyNAMiC investigates individual differences in rates of changes in brain and cognitive measures, we estimated the power to detect individual changes in cognition (McArdle & Nesselroade, 1994). We considered a latent difference model where, for each time point, latent overall cognition is measured by latent domains of episodic memory, working memory, and perceptual speed. Each latent domain, in turn, is measured by three manifest variables that correspond to the tests within the cognitive test battery. We computed the power to detect longitudinal changes in overall cognition for a set of model parameters, such as the reliability of measures, attrition rate, and variance of longitudinal change. Further details of the power analyses are given in the Supporting Information.

In the simulations, the power to detect longitudinal change was approximated to be at least 88% when at least 80% reliability was considered (the reliability of most cognitive tests in DyNAMiC was estimated to be around 0.8–0.95) and variance of change was considered to be at least 20% of the baseline variance (in a similar imaging sample, Betula: Nilsson et al., 2004), the variance of longitudinal change was around 42% of the variance in initial level.

## 3 RESULTS

### 3.1 Sample characteristics

Demographic information from the DyNAMiC sample is presented in Table 1. The educational level of the sample was relatively high, with 58.4% of participants reporting university‐level education. This is consistent with Umeå being one of Sweden’s main university cities, similarly reflected in the educational levels in other Umeå‐based study samples (Nevalainen et al., 2015; Nilsson et al., 1997). Only a small group of participants were unemployed (3.9% of the full sample), while a majority of the sample reported some form of employment (69.1%), and a majority of older individuals (> 60 years) had retired (74.6%). Although the average number of children across the full sample (1 ± 1.9) was lower than the national figure of 1.7 (Statistics Sweden, 2019, https://www.scb.se/en/), numbers observed for individuals >40 years were higher (means ranging between 1.8 and 2.3 across age groups).

An overview of health parameters is presented in Table 2. Examination of medical information shows that 50% of participants reported using some form of medication. The most common treatment was for hypertension, reported by 15.7% of participants, with a majority of these participants (78.6%) belonging to the two oldest age groups (>60 years). The prevalence of hypertension medication was overall 34.9% in participants over the age of 60. The second most common medical treatment was for asthma (8% of the sample), followed by hyperlipidemia (7%), and cardiovascular disease (6%). These observations indicate that treatments regulating cardiovascular disease and risk factors, such as high blood pressure and cholesterol, were most prevalent overall, with numbers driven by the older segment of the sample. Average BMI values (ranging from 24.3 to 26.7 across age groups) were within the normal to overweight span (19–30). In total, 19.6% of participants reported consuming nicotine (smoking and/or using snus), with the largest number of smokers found in the youngest (*n* = 6) and oldest (*n* = 4) groups.

### 3.2 Cognitive performance

**FIGURE 3:**
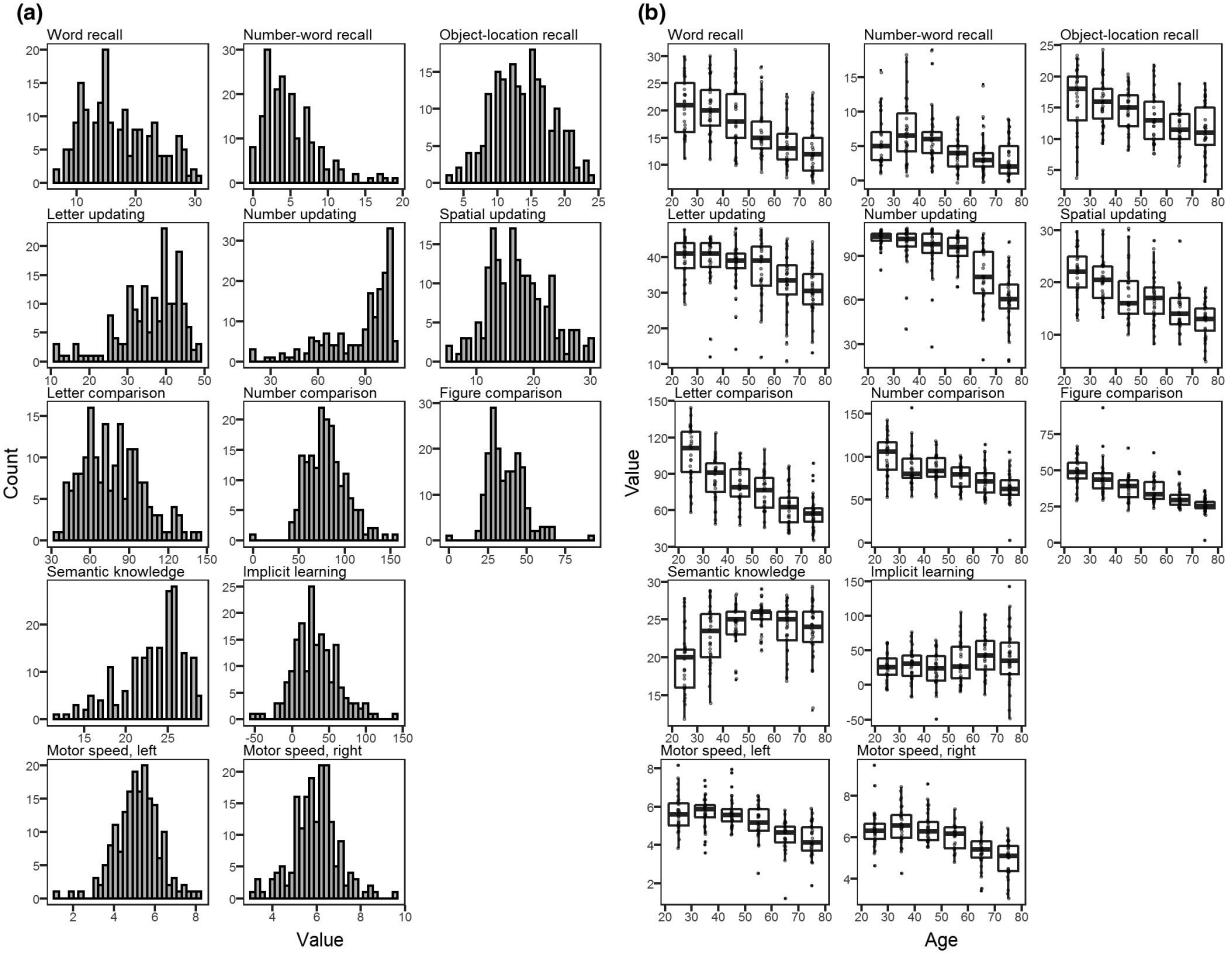
Cognitive performance. (a) Histograms showing distribution of test scores for each cognitive task. (b) Boxplots of cognitive test scores stratified by age

Distributions of responses across cognitive tests are presented in Figure 3a. Results of normality tests are presented in the Supporting Information. The distribution of scores from the episodic number‐word recall test indicated that this task was difficult for participants to perform. The working memory number‐updating task, on the other hand, had a high proportion of high scores from younger individuals (see Figure 3b), whereas most of the low scores in this task came from the older subjects. Overall, lowest performances were observed in older participants, except for the vocabulary and implicit learning tests (Figure 3b).

#### 3.2.1 The factor structure of episodic memory, working memory, and perceptual speed

Given relatively different distributions of scores across cognitive tests, it is reasonable to expect that these tests might differentially represent their respective cognitive domains.

To validate the contribution of the considered cognitive domains to the observed data, the structure of the episodic memory, working memory, and perceptual speed was assessed through structural equation modeling (SEM). We investigated a similar latent structure to the one reported in the COBRA study that had the same test battery (Nevalainen et al., 2015) to relate the latent structure in our age‐heterogeneous sample to the latent structure in COBRA’s age‐homogeneous sample. Out of 180 subjects, one participant had the spatial‐updating task score missing, and one subject had the scores for all working memory tasks missing. Before fitting a SEM model, we excluded univariate outliers, that is the observations with the absolute value of the standardized score greater than 3.29 (the probability to observe such values when the data are normally distributed is <0.001). In total, five such univariate outliers were detected: two observations for number‐word recall, two observations for number comparison, and one observation for figure comparison. We then standardized the scores for all the tests and age and deleted five observations that were detected as bivariate outliers based on the Mahalanobis distance calculated for each pair of observed test scores within each cognitive domain (*χ* ^2^(2) > 13.82, *p* < 0.001, based on the data from 180 subjects without five univariate outliers). Bivariate outlying observations included scores for all episodic memory tests for one subject, and scores for the letter‐updating and the number‐updating task for another person. After deleting bivariate outliers, no observations were detected as trivariate outliers based on the scores for each cognitive domain separately. The univariate and bivariate outlying observations were excluded from the analysis. All other existing data for the subjects with some outlying or missing observations were used in the estimation. To investigate the relationships between the cognitive domains that are not driven by age heterogeneity of our sample, we included age as a covariate in the model. Estimation using full information maximum likelihood was performed in AMOS 26.0.0 (Arbuckle, 2019).

**FIGURE 4:**
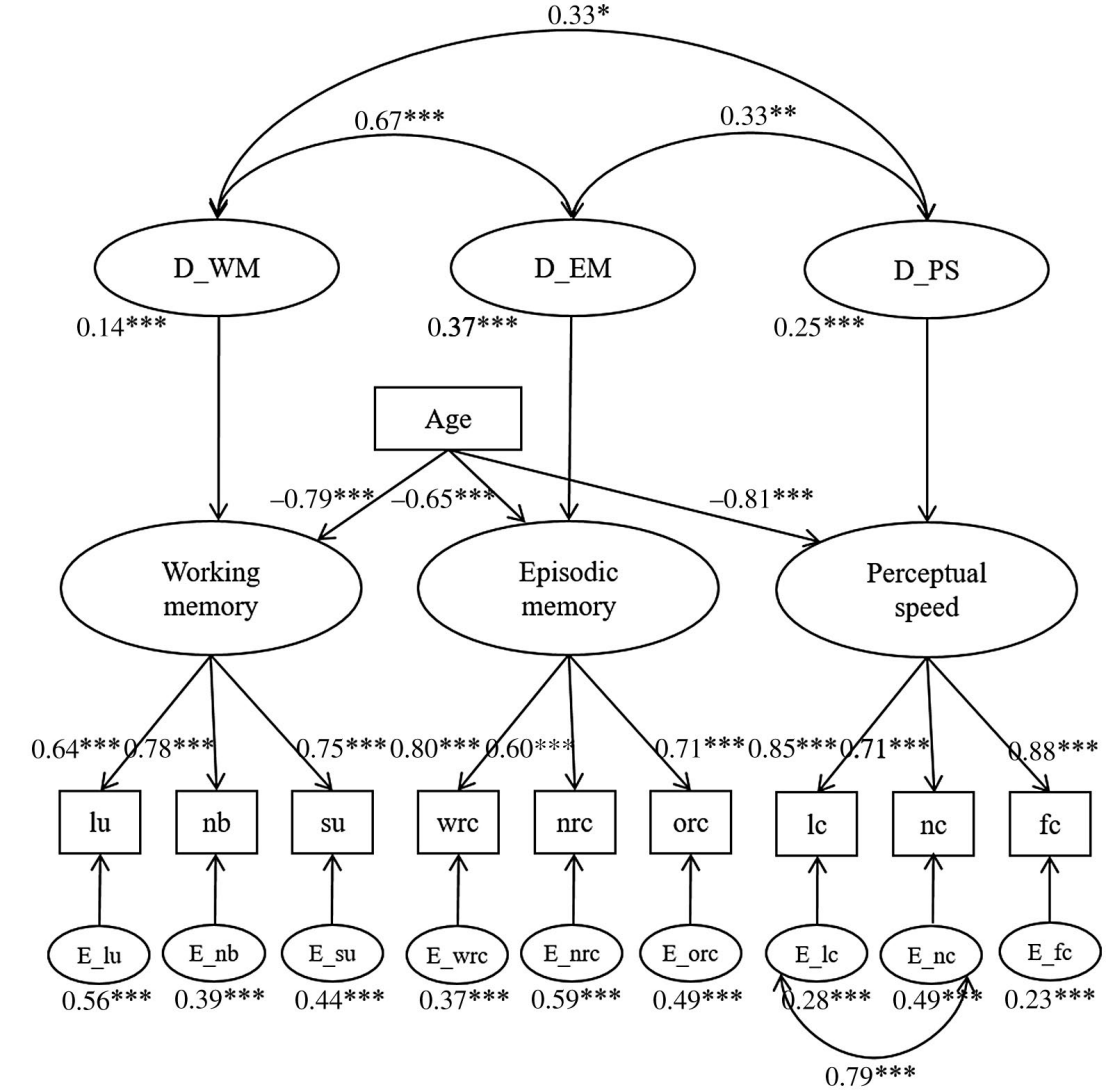
Structural‐equation model including factors of episodic memory (wrc, word recall; nrc, number‐word recall; orc, object‐location recall), working memory (lu, letter updating; nb, number updating; su, spatial updating), and perceptual speed (lc, letter comparison; nc, number comparison; fc, figure comparison). The figure provides correlations between the latent variables, standardized regression weights, as well as estimated variances of latent variables. *p < 0.05, **p < 0.01, ***p < 0.001 in *z*‐tests for model parameters

The model had a good fit to the data (*χ* ^2^ = 36.326, *df* = 29, *n* = 180, *p* = 0.164, RMSEA = 0.038 (90% confidence interval = [0, 0.072], CFI = 0.993), which suggested the existence of latent variables corresponding to the three cognitive domains: episodic memory, working memory, and perceptual speed (Figure 4). Similar to the COBRA study (Nevalainen et al., 2015), that used the same test battery to investigate cognition within a limited age span (64–68 years), the correlation between working and episodic memory cognitive domains was the strongest.

### 3.3 Dopamine receptor binding potential (BP_ND_ )

#### 3.3.1 Dopamine D1DR

**FIGURE 5:**
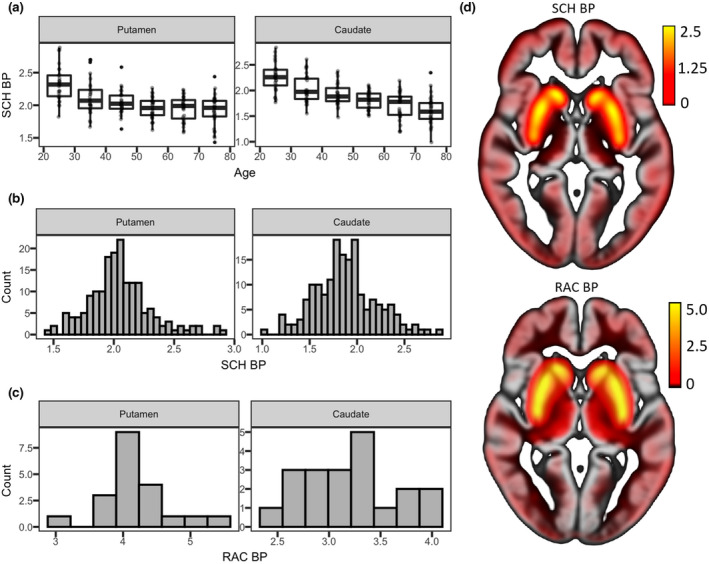
Dopamine receptor BP_ND_ (PVE corrected) for the putamen and caudate nucleus. (a) Regional distributions of D1DR BP_ND_. (b) D1DR BP_ND_ across age groups. (c) Regional distributions of D2DR BP_ND_ in the older subsample of participants (*n* = 20, >65 years). (d) Voxel‐wise BP_ND_ estimates overlaid on a sample‐specific gray matter template in Montreal Neurological Institute (MNI) space. Average maps of *n* = 20 older participants scanned using both radioligands are displayed

Distributions of regional [^11^C]SCH23390 BP_ND_ estimates (PVE‐corrected) are presented in Figure 5a. No evidence for non‐normality was observed in the caudate nucleus (Shapiro–Wilks test; W = 0.99; *n* = 176; *p* = 0.29), whereas the distribution of putamen BP_ND_ was positively skewed (W = 0.97; *n* = 176; *p* < 0.001; skewness = 0.64, kurtosis = 4.08). Age‐stratified boxplots presented in Figure 5b did not indicate outlier observations outside a biologically plausible range, and showed that the youngest participants tended to exhibit particularly high BP_ND_s in the putamen. Hence, it appears likely that skewness in BP_ND_ distributions was related to the large age span in the present cohort. Highest average BP_ND_s were observed in the putamen (2.05 ± 0.26), followed by the caudate nucleus (1.88 ± 0.33), replicating the rank order of BP_ND_s reported in autopsy work (Hall et al., 1994).

#### 3.3.2 Dopamine D2DR

Distributions of regional [^11^C]raclopride BP_ND_ estimates (PVE corrected) are presented in Figure 5c. The small size of the subsample exposed to [^11^C]raclopride‐PET imaging did not allow statistical evaluation of the distributions. Rank order of regional BP_ND_s was, however, in good concordance with earlier studies (Hall et al., 1994; Papenberg et al., 2019). Highest average BP_ND_s were observed in the putamen (4.17 ± 0.51), followed by the caudate nucleus (3.20 ± 0.43).

### 3.4 Association of D1DR and functional connectivity across cortical regions

**FIGURE 6:**
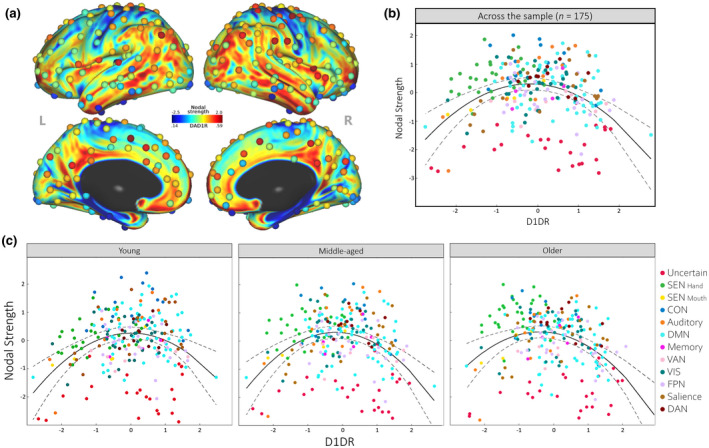
Associations between cortical D1DR and functional nodal strength. (a) Surface projection of average cortical D1DR in 32k MSMAll HCP surface space (Glasser et al., 2016) and cortical nodes (color coded by nodal strength) in the Power atlas (Power et al., 2011). (b) Inverted u‐shaped association between cortical D1DR and nodal strength (bold line: quadratic fit; dashed line: 95% CI). Stepwise modeling of linear and quadratic effects across the sample (*n* = 175) revealed that a quadratic model (*R* ^2^ = 0.14; RMSE = 3.96; LogLik. = −677.51) explained an additional 14.9% variance (likelihood‐ratio test, *p* < 0.001) compared to a linear model (*R* ^2^ = −0.0028; RMSE = 4.28; LogLik. = −697.14). (c) Quadratic fit of cortical D1DR on nodal strength within young, middle‐aged, and older age groups. Colors of cortical nodes indicate nodal network designation (red, uncertain; light green, sensorimotor hand; yellow, sensorimotor mouth; dark blue, cingulo‐opercular; orange, auditory; light blue, default mode; dark pink, memory retrieval; light pink, ventral attention; dark green, visual; light purple, fronto‐parietal; brown, salience; burgundy, dorsal attention)

The distribution of regional D1DR across the cortex was found to slightly deviate from normality (Shapiro–Wilks test, W = 0.988; *n* = 243; *p* = 0.04), whereas the distribution of cortical nodal strength was negatively skewed (W = 0.963; *n* = 243; *p* < 0.001; skewness = −0.699; kurtosis = 3.278). The spatial distribution of D1DR across cortex and functional nodes is displayed in Figure 6a. Stepwise modeling of linear and quadratic effects of D1DR on nodal strength across the sample revealed that a quadratic model (*R* ^2^ = 0.14; RMSE = 3.96; LogLik. = −677.51) explained an additional 14.9% variance (likelihood‐ratio test, *p* < 0.001), compared to a linear model (*R* ^2^ = −0.0028; RMSE = 4.28; LogLik. = −697.14). The quadratic model conveyed an inverted u‐shaped effect of D1DR on nodal strength across regions of cortical networks (Figure 6b). This suggests a level‐dependent modulation of the functional connectome at a global level by the DA D1 system, where the greatest nodal strength is observed in regions with intermediate levels of D1DR. Similar quadratic associations were observed within each age decade. Given comparable results within each decade, we combined the smaller age groups into three larger groupings for young, middle‐aged, and older adults. For all three groups, the quadratic model (young: *R* ^2^ = 0.12; RMSE = 0.94; LogLik. = −328.35; middle‐aged: *R* ^2^ = 0.14; RMSE = 0.93; LogLik. = −325.05; older adults: *R* ^2^ = 0.15; RMSE = 0.92; LogLik. = −324.03) significantly outperformed (young: likelihood‐ratio test, *p* < 0.001; middle‐aged: likelihood‐ratio test, *p* < 0.001; older adults: likelihood‐ratio test, *p* < 0.001) the linear version (young: *R* ^2^ = 0.009; RMSE = 0.99; LogLik. = −342.69; middle‐aged: *R* ^2^ = −0.002; RMSE = 1.01; LogLik. = −344.06; older adults: *R* ^2^ = 0.03; RMSE = 0.99; LogLik. = −340.22), suggesting a robustness to the inverted u‐shaped effect of regional cortical D1DR on connectivity across the lifespan (Figure 6c).

## 4 DISCUSSION

This paper introduced the DyNAMiC study, designed to meet the current paucity of longitudinal multimodal data necessary for a comprehensive understanding of age‐related changes in cognition across the adult lifespan. It is well documented that cross‐sectional and longitudinal estimates of age‐related changes in brain integrity and cognition deviate (Fjell et al., 2015; Nyberg et al., 2010; Raz et al., 2005; Rönnlund et al., 2005; Salthouse, 2010). Critically, only a few studies have explored longitudinal changes in the functional connectome (Chong et al., 2019; Malagurski et al., 2020; Ng et al., 2016; Pedersen et al., 2021), whereas longitudinal estimates of D1DR—one of the most age‐sensitive DA receptor systems—are entirely missing. Moreover, studies investigating the link between DA neurotransmission and the functional connectome, and its potential impact on cognition in aging, are lacking in both cross‐sectional and longitudinal settings. To address these unresolved issues, DyNAMiC investigates changes in the brain’s connectome and D1DR, examines their associations, and how alterations in these measures map onto cognitive changes in aging. In the current study, we provide a detailed characterization of DyNAMiC, and initial results linking the brain’s functional connectome to D1DR across the adult lifespan.

DyNAMiC includes individuals from six decades of the adult human lifespan (*n* = 180, 20–79 years). Considering sex as an important biological variable, efforts were made to achieve even distributions of men and women in the sample, and within each age cohort. While the current study did not test hypotheses of sex differences, future studies are well‐resourced to assess potential effects of sex across the adult lifespan. Despite implementation of various exclusion criteria during recruitment, some medical conditions were reported by participants after study inclusion, resulting in a higher than anticipated proportion of participants with medical treatment (Table 2). The prevalence of hypertension medication was overall 35% in participants over the age of 60 (who made up 78.6% of reported cases in the sample), corresponding well to estimates for other Swedish samples: 45% in individuals 25–64 years (Persson et al., 2002); ~50% in 60‐year olds (Carlsson et al., 2008); and 33% in adults 64–68 years, also recruited from Umeå (Nevalainen et al., 2015). The level of education was higher in DyNAMiC (58.4%) compared to the Swedish national average (44%: Statistics Sweden, 2019, https://www.scb.se/en/), consistent with Umeå being one of the major university cities in Sweden.

To create multifaceted measures of episodic memory, working memory, and perceptual speed, each domain was tested using three tests, including verbal, numerical, and figural materials, respectively. The low scores on episodic number‐word recall suggest that this test was difficult for participants to perform, consistent with results from a previous study using the same test battery (Nevalainen et al., 2015). In contrast, a large proportion of participants (age < 55 years) displayed high scores on the working memory number‐updating test, indicating that this was not a very challenging task. Given these observations, we assessed the factor structure of the episodic memory, working memory, and perceptual speed tests through SEM, evaluating the contribution of each subtest to its corresponding domain. Modeling suggested the existence of three latent cognitive domains demonstrating shared variance across subtests (Figure 4). Given that SEM capitalizes on the shared variance across subtests, it is conceivable that using factor scores from such a model provides a good alternative to mean‐based composite measures of performance. Importantly, significant negative associations with age were evident for all three cognitive domains, in line with previous literature indicating their age sensitivity (Rönnlund et al., 2005; Salthouse, 2010). In sum, modeling of cognitive performance indicated that the first wave of DyNAMiC provides the means to examine diverse and complex aspects of cognition in aging. Moreover, power analyses suggested that the power to detect longitudinal changes in cognition from DyNAMiC data is expected to be high.

DA modulation of synaptic activity enhances specificity in neuronal signal (Seamans & Yang, 2004; Shafiei et al., 2019; Shine et al., 2019), and past studies have reported associations of D1DR with brain activation and FC in young adults (Roffman et al., 2016; Turner et al., 2020). As such, age‐related DA decline might constitute a basis for changes in the functional connectome. Effects of DA on FC within large‐scale brain networks are, however, reported as diverse (Cole et al., 2013; Wallace et al., 2011), although previous studies indicate regional variability in the association between DA and FC (Tang et al., 2019; Xu et al., 2016), and that the spatial distribution of neurotransmitter receptors contribute to the brain’s functional architecture (Dukart et al., 2021; Hansen et al., 2021). This may have important implications for the functional connectome in aging, as indicated by a recent study showing that regional variability in age‐related effects on FC was related to D1DR (Garzón et al., 2021). However, given that findings on regional variability in the association between D1DR and FC at a systems level are sparse, we tested the link between D1DR and FC across cortical regions corresponding to nodes in large‐scale functional networks. Initial analyses indeed demonstrated significant variation in FC across cortical nodes as a function of D1DR, conveyed by a non‐linear association such that regions with the lowest and highest levels of D1DR displayed the lowest nodal strength, whereas the highest nodal strength was evident for regions at an intermediate level of D1DR. This association was consistent across age groups, suggesting that the configuration of functional regions depend on underlying receptor distribution (Shine et al., 2019). Moreover, our results suggest that age‐related D1DR loss could be associated with diverse, region‐dependent, differences in FC. In turn, the impact of altered D1DR levels on cognition might vary across cognitive domains, to the extent that specific cognitive functions differentially depend on distributed regions.

However, although the non‐linear association between D1DR and FC observed across cortical regions mirrors the well‐established inverted u‐shaped effect of DA on cognition, where both excessive and insufficient levels of DA are deleterious for cognitive function (Cools & D’Esposito, 2011; Zahrt et al., 1997), it is important to note that our observation is not based on inter‐individual differences, but rather D1DR and FC across different regions. As such, further examination is needed to explore this non‐linear D1DR‐FC association across cortical network nodes in relation to individual differences in these measures. Given previous cross‐sectional observations of age‐related D1DR decline (Karrer et al., 2017), healthy aging might be accompanied by levels of D1DR occupancy outside the optimal range for efficient neural signaling, similar to other conditions characterized by D1DR deficiency (e.g., Parkinson’s disease; Goldman‐Rakic et al., 2000). This, in turn, may result in impaired cognitive function (Li et al., 2010; Lindenberger et al., 2008). Relatedly, degeneration within the mesocorticolimbic DA system has been implicated in cognitive decline and disease progression in Alzheimer’s disease (Martorana & Koch, 2014; Trillo et al., 2013). Taken together, aberrant DA‐FC associations may as such serve as a potential marker of cognitive decline in aging, and of older individuals at risk of converting to pathological aging.

Although SEM conveyed significant negative associations between cognitive domains and age, it is important to note that the current study on baseline data cannot characterize inter‐individual differences in age‐related trajectories of cognitive and brain measures. Instead, the main aim was to provide a comprehensive descriptive characterization of DyNAMiC baseline data, in parallel to assessing potential links between select core measures, which can be further explored in upcoming investigations. As such, whereas the stratification of participants into age groups does not capture the full extent and qualities of possible age‐related effects, it reflects the lifespan design of the DyNAMiC sample. Due to the strict recruitment criteria, older DyNAMiC participants are considerably healthy, making it likely that a proportion of those elderly individuals are so called super agers (Harrison et al., 2012; Pudas et al., 2013; Yu et al., 2020), who throughout aging beyond 80 years display characteristics on par with those observed in 50–60 year olds (Nyberg et al., 2020). This may, already at baseline, be reflected in higher than expected levels of cognitive function and brain integrity, in turn attenuating effects of age. For instance, given that episodic memory is a highly age‐sensitive domain (Gorbach et al., 2017; Rönnlund et al., 2005; Schaie, 1994), initial observations of small differences between age groups might indeed suggest an impact of the older participants' good health status.

Some challenges are associated with the design of DyNAMiC. For instance, effects of attrition risk biasing longitudinal estimates of change (Eisner et al., 2019; Goodman & Blum, 1996; Lewin et al., 2018), but are an almost inevitable feature of longitudinal studies due to factors such as relocation, mortality, and arising MRI incompatibility. Attrition is often meaningful and non‐ignorable, given significant associations of drop‐out status and decline in brain and cognitive integrity (Josefsson et al., 2012; Nyberg et al., 2019; Nyberg & Pudas, 2019). Even though careful means were taken to achieve a healthy sample, it is indeed possible that some participants will convert from normal to pathological aging over time, which might affect several aspects of the brain—for instance the functional connectome (Filippi et al., 2020; Fox & Greicius, 2010; Sheline & Raichle, 2013; Zhang & Raichle, 2010). Accounting for dependencies between attrition and variables of interest will therefore be important in identifying reliable change–change associations between brain integrity and cognition (Gorbach et al., 2017; Josefsson et al., 2012; Little, 1995).

Finally, DyNAMiC does not include the oldest‐old individuals (>80 years), in contrast to other longitudinal and multimodal brain imaging initiatives like the Human Connectome Project in Aging (Bookheimer et al., 2019), and the Umeå‐based Betula study (Nilsson et al., 1997, 2004). This demographic is in Sweden expected to increase by 50% between 2018 and 2028 (Statistics Sweden, 2019, https://www.scb.se/en/), but remains an age segment left out of most imaging studies to date. At the second time point, DyNAMiC will, however, be able to include returnees over the age of 80, providing information on potential changes during these individuals' transition into the oldest‐old demographic.

## 5 CONCLUSIONS

The first wave of DyNAMiC has provided a large multimodal data set, which will advance our understanding of lifespan alterations in human brain structure, function, and DA neurotransmission, as related to each other and to cognitive decline in aging. DyNAMiC is the largest DA D1 study worldwide, and will be able to examine trajectories and rates of change; identify onsets of brain and cognitive decline informing the optimal time point for interventions; tease apart shared and unique contributions of different brain measures to changes in cognition; and identify associations between functional and molecular brain systems as potential mechanisms of cognitive decline. Initial observations indicated that spatial configuration of functional regions depends on underlying DA receptor distribution, and for the first time revealed a non‐linear effect of D1DR at a global neuronal level across the adult lifespan.

### DECLARATION OF TRANSPARENCY

The authors, reviewers and editors affirm that in accordance to the policies set by the *Journal of Neuroscience Research*, this manuscript presents an accurate and transparent account of the study being reported and that all critical details describing the methods and results are present.

## CONFLICT OF INTEREST

The authors declare no conflict of interest.

## ETHICS APPROVAL

This study was approved by the Regional Ethical board and the local Radiation Safety Committee in Umeå, Sweden.

## AUTHOR CONTRIBUTIONS

*Conceptualization*, A.S.; *Data Curation*, M.A., R.P., and V.P.L.; *Formal Analysis*, T.G., R.P., M.A., and J.J.; *Funding Acquisition*, A.S.; *Investigation*, K.N., V.P.L, and R.P.; *Methodology*, A.S., L.B., G.K., G.P., and A.W.; *Project Administration*, K.N., T.G., and A.S.; *Resources*, K.R.; *Visualization*, V.P.L, R.P., T.G., and J.J.; *Writing – Original Draft*, K.N., T.G., A.S., V.P.L., R.P., and J.J.; *Writing – Review & Editing*, K.N., T.G., A.S., V.P.L., R.P., J.J., C.M., K.R., G.P., A.W., G.K., and L.B.

### PEER REVIEW

The peer review history for this article is available at https://publons.com/publon/10.1002/jnr.25039.

## Supporting information


**TABLE S1** Participants by decade and sex. Completed participants have full data from the study components. The age range for the double PET subsample was 65–79. Dropouts completed the first study session (MRI), but not the second (PET)
**TABLE S2** Number of invitations sent out during recruitment, for men and women within each decade
**TABLE S3** Social, cognitive, and physical activities (mean number of hours per week ± standard deviation)
**FIGURE S1** (a) Histograms portraying distributions of adjusted regional gray matter volumes (cm^3^) for the putamen, caudate nucleus, nucleus accumbens, cingulate cortex, frontal lobe, and hippocampus. (b) Adjusted gray matter volumes across age groups

Transparent Science Questionnaire for Authors

## Data Availability

Data from the DyNAMiC project cannot be made publicly available due to ethical and legal restrictions. However, access to these original data may be available upon request from the corresponding author.
