# Supplementary material for: DyNAMiC: A prospective longitudinal study of dopamine and brain connectomes: A new window into cognitive aging
Source: J Neurosci Res. 2022 Mar 16;100(6):1296–320. doi: 10.1002/jnr.25039 (PMC9313590; doi:10.1002/jnr.25039)
Supplement: Supplementary file 1 — TABLE S1 Participants by decade and sex. Completed participants have full data from the study components. The age range for the double PET subsample was 65–79. Dropouts completed the first study session (MRI), but not the second (PET) TABLE S2 Number of invitations sent out during recruitment, for men and women within each decade TABLE S3 Social, cognitive, and physical activities (mean number of hours per week ± standard deviation) FIGURE S1 (a) Histograms portraying distributions of adjusted regional gray matter volumes (cm3) for the putamen, caudate nucleus, nucleus accumbens, cingulate cortex, frontal lobe, and hippocampus. (b) Adjusted gray matter volumes across age groups [file JNR-100-1296-s001.docx]

**S.1. Recruitment procedure**

Supplementary Table 1. *Participants by decade and sex. Completed participants have full data from the study components. The age range for the double PET subsample was 65-79. Dropouts completed the first study session (MRI), but not the second (PET).*

| AGE  GROUP | COMPLETED | | | | DROPOUTS | | TOTAL | |
| --- | --- | --- | --- | --- | --- | --- | --- | --- |
|  | Single PET | | Double PET | |  |  |  |  |
|  | ♀ | ♂ | ♀ | ♂ | ♀ | ♂ | ♀ | ♂ |
| 20-29 | 13 | 15 |  |  | 1 | 0 | 14 | 15 |
| 30-39 | 15 | 14 |  |  | 1 | 0 | 16 | 14 |
| 40-49 | 14 | 15 |  |  | 0 | 0 | 14 | 15 |
| 50-59 | 14 | 15 |  |  | 0 | 0 | 14 | 15 |
| 60-69 | 12 | 13 | 3 | 2 | 0 | 0 | 15 | 15 |
| 70-79 | 9 | 8 | 7 | 8 | 1 | 0 | 17 | 16 |
| TOTAL | 77 | 80 | 10 | 10 | 3 | 0 | 90 | 90 |
|  | 157 | | 20 | | 3 | | 180 | |

To achieve a full study sample, a total of 2237 invitation letters were sent out across several rounds (Supplementary Table 2). In total, there were over 400 responses from interested individuals. Eligible candidates were, after an initial screening, included in the order that they made contact upon invitation. In case an eligible candidate’s age group was already full, they were listed as a potential replacement in case an included participant was to drop out before testing. In case of exclusion, the individual was informed, the reason for exclusion was annotated anonymously, and all documents containing personal information were destroyed. In total, 154 individuals (~38 % of the respondents) were excluded from participating in the study. The most common reasons for exclusion were psychopharmacological treatment (n=42; 27.3%), psychiatric diagnosis (n=41; 26.6%), metal implants incompatible with MRI examination (n=23; 14.9%), and radiation safety contraindications (n=23; 14.9%). Brain injury or neurological disorder was reported by 21 individuals (13.6%), diabetes by 18 (11.7%), severe hearing loss by 14 (9.1%), and non-native proficiency in Swedish by 14 (9.1%).

Supplementary Table 2. *Number of invitations sent out during recruitment, for men and women within each decade.*

| AGE GROUP | FIRST ROUND | | SECOND ROUND | | ADDITIONAL | | TOTAL | |
| --- | --- | --- | --- | --- | --- | --- | --- | --- |
|  | ♀ | ♂ | ♀ | ♂ | ♀ | ♂ | ♀ | ♂ |
| 20-29 | 70 | 70 | 70 | 70 | 109 | 8 | 249 | 148 |
| 30-39 | 70 | 70 | 70 | 70 | 106 | 34 | 246 | 174 |
| 40-49 | 70 | 70 | 70 | 70 | 59 | 64 | 199 | 204 |
| 50-59 | 70 | 70 | 70 | 70 | 14 | 13 | 154 | 153 |
| 60-69 | 70 | 70 | 70 | 70 | 39 | 24 | 179 | 164 |
| 70-79 | 70 | 70 | 70 | 70 | 51 | 34 | 191 | 174 |

**S.2. Statistical power**

Because DyNAMiC investigates individual differences in rates of changes in brain and cognitive measures, we estimated the power of the latent difference model in detecting individual changes in cognitive measures (McArdle & Nesselroade, 1994). In so doing, for each set of parameters, we simulated 100 replications of data from the latent change model as described below and fitted this model using the lavaan package (Rosseel, 2012) in R software (R Core Team, 2018). The analyses are made available online (<https://github.com/tetianagorbach/DyNAMiC-introduction/tree/master/Power_estimation>). Estimates of power were then calculated as the proportion of replications in which the variance in change was estimated to be significantly different from zero. In the calculations, we used a sample size of 180 and 2 measurement waves. Attrition rates were 10%, 20%, and 40%. The model had two levels of latent variables at each wave.

The top-level latent variables included only a latent variable for overall cognition at baseline, latent cognitive change, and latent overall cognition at follow-up. Overall cognition at the follow-up is defined as the sum of baseline and change measures. The mean of the overall cognition at the first wave was set to 50, and the variance was set to 1. The mean of latent change was 5, and the variance was 10%, 20%, and 50% of the initial level variance. The self-feedback parameter (correlation between change and overall cognition at the baseline) was set to -0.05, -0.2.

The top-level cognition is measured by the second level latent variables, such that each wave had three latent variables representing three cognitive domains (episodic memory, working memory, and perceptual speed). The latent variables for the cognitive domains did not have intercepts. According to the project’s testing procedures, each of the latent variables for the cognitive domains were measured by three manifest variables. All regression coefficients in the measurement part of the model were set to 1. Random errors (in the measurement model and disturbances for cognitive domains) were generated as a multivariate normal random variable (18 errors for three observed variables per three cognitive domains and two waves plus six errors for each of the three cognitive domains and two waves). The variance of errors was defined so that the reliability of each observed score and latent variable for the cognitive domains (ratio between the variance of an observed test and the true latent construct affecting it) was 0.7, 0.8, 0.9, or 0.95.

The attrition rate, self-feedback, the variance of change of the overall cognition, and the reliability of measures, varied between different sets of model parameters. The reliability and the variance of change affected the quality of estimation as well as the power. When the variance of latent change increased from 10% to 50% of the initial-level variance, the average power (across the parameters) increased from 63% to 99.8%. A similar increase from 68% to 100% was observed when the reliability of measures changed from 0.7 to 0.95. Other factors, i.e., attrition rate and self-feedback, did not significantly affect the power of estimation.

Numerical optimizations for all simulations converged; however, when the reliability or the true variance of change was small, the variance of change was sometimes estimated as negative. This happened, for example, in 20% of replications on average when the reliability was 0.7 and variance of change was equal to 10% of the baseline variance, but only occurred twice out of 7200 replications when the variance of change was at least 20% of the initial variance and the reliability was at least 0.8. The reliability of most cognitive tests in DyNAMiC was estimated to lie around 0.8-0.95, and we expect considerable variance of longitudinal change, because in a similar imaging sample (Betula: Nilsson et al., 2004), the variance of longitudinal change was around 42% of the variance in initial level. Therefore, we do not expect the variance of change to be estimated as negative in DyNAMiC.

Based on the simulations, if the reliability of measures is at least 80%, and the variance of change is at least 20% of the baseline variance, then the power to detect longitudinal change is at least 88%.

**S.3. Functional MRI**

Since GE has recently developed a research-based sequence for multiband fMRI, we collected 10 subjects with both multiband and standard resting-state fMRI, to examine the quality of the multiband sequence and compare it with the standard sequence (results presented in master’s thesis: Björnfot, 2018; <https://www.diva-portal.org/smash/record.jsf?pid=diva2:1228907>). This was a resting-state single-shot multiband EPI sequence of 1110 volumes acquired over 12 minutes. The total slice number was 44 with a slice thickness of 3 mm, TR = 650 ms, TE = 30 ms, flip angle = 60º, TR = 650 ms, FOV = 240 x 240 mm, and matrix size = 80 x 80. The multiband-acceleration factor was 6, with no in-plane acceleration. Two sets of ten *b* = 0 baseline images were collected with opposing polarities in the phase encoding direction, to correct for spatial distortions due to susceptibility-induced magnetic field inhomogeneities.

*The functional connectome at different mental states*

The functional connectome displays both trait- and state-dependent components, with trait-like aspects of FC remarkably similar across various cognitive domains (Cole et al., 2014), whereas other aspects of FC vary across mental states (Avelar-Pereira et al., 2017; Geerligs et al., 2015). Further, individual differences in FC (e.g., due to aging), and its ability to predict cognition may be enhanced during task versus rest (Greene et al., 2018). Currently, it remains unclear whether the link between FC and cognition, and their longitudinal associations, vary across mental states.

In addition to the resting-state fMRI condition described in the main text, whole-brain functional images were acquired during conditions of naturalistic viewing and working-memory, allowing for estimation of the functional connectome at different mental states. Images for the naturalistic viewing condition were sampled using the same sequence as for the resting-state condition. The working memory *n*-back task sequence was slightly shorter with 330 volumes collected over 11.3 minutes. All functional sequences were sampled with 37 transaxial slices, slice thickness = 3.4 mm, 0.5 mm spacing, TR = 2000 ms, TE = 30 ms, flip angle = 80º, and FOV = 250 x 250 mm. Ten dummy scans were collected at the start of each sequence.

Working memory was assessed in the scanner with a numerical *n*-back task (Salami et al., 2018, 2019) implemented in E-prime (Psychology Software Tools). This task was selected based on correspondence to the COBRA data set (Nevalainen et al., 2015), enabling comparisons and pooling of data. Further, previous findings indicate that different levels of working memory load provide a suitable setting for examining DA-dependent modulation of brain activation (Salami et al., 2019). This task required participants to maintain and update information in memory, for each presented item deciding whether it matched an item presented a certain number of steps (*n* steps) back. A series of single digit numbers between 1 and 9 were displayed for 1.5 s in the center of the monitor, separated by a fixation cross for 0.5 s. The task involved three conditions, 1-, 2-, and 3-back, corresponding to successively increasing working-memory load. Each condition included 9 blocks of 10 numbers, presented in random order. Before the start of each block, participants were presented with a picture cueing the type of block about to start. Participants gave their responses using two buttons on a MRI-compatible response device, which they were familiarized with prior to scanning. The right index finger was used for responding “yes, it is the same number” and the right middle finger was used for responding “no, it is not the same number”. Every correct response (yes and no) gave 1 point and was used in calculating performance. Responses to the first item in 1-back blocks, and the 1-2, and 1-3 items in 2-back and 3-back blocks, were not included in the total score. The maximum score for the three conditions was 81, 72 and 63, respectively. All participants completed a minimum of 2 practice trials of the task prior to entering the scanner, but were allowed as many repetitions as needed, to understand the instructions and the response buttons.

Naturalistic viewing, previously suggested to affect individual variation in FC (Finn et al., 2017; Hasson et al., 2010; Vanderwal et al., 2017), constitutes an intermediate state in relation to the constrained task condition and the unconstrained resting-state condition. During this condition, participants viewed and listened to a 12-minute video consisting of selected and chronologically ordered sections from the Swedish movie “Cockpit”. Participants were instructed to view the movie attentively and answered a short multiple-choice questionnaire about the movie after the scanning session.

**S.4. Sample characteristics: social, cognitive, and physical activities**

Supplementary Table 3 provides an overview of participants’ self-rated activity levels within social activities (e.g. spending time with family and friends; visiting restaurants and cafés; going to parties), cognitive activities (e.g. reading; writing; playing an instrument), and physical activities (e.g. walking; jogging; doing sports). Frequency of an activity was measured as hours per week (ranging from 0 to >15 hours) during a typical summer week. Following questions on cognitive and physical activities, participants rated how demanding, on a scale from 0 to 5, they normally found these activities to be.

The social activities ranking highest in the number of hours per week were spending time with family members (10.7±5.1) and friends (4.9±3.8). Cognitive activities rated as most frequent were using the computer for purposes other than games (5.9±5.1), cooking (5.8±3.4), and driving (4.2±3.7), with writing texts in fourth place (3.0±3.5). Walking (5.7±3.7) was the highest rated physical activity, followed by cycling (3.3±3.4) and gardening (3.0±3.7). Some activities were rated as most frequent by young individuals (< 40 years). These include spending time with family and friends; going to restaurants/pubs/cafés and social gatherings; playing computer games; engaging in educational activities such as lectures, reading non-fiction, and studying at home; as well as doing different types of workout and sports. In contrast, older adults (>60) reported the highest frequencies of all age groups for talking on the phone with family members, relatives, and friends; reading novels and newspapers; solving crossword puzzles; engaging in gardening, fishing, and picking mushrooms.

Supplementary Table 3. *Social, cognitive, and physical activities (mean number of hours per week ± standard deviation).*

|  |  |  |  |  |  |  |  |  |  |
| --- | --- | --- | --- | --- | --- | --- | --- | --- | --- |
| ACTIVITY | TOTAL SAMPLE | AGE  20-29 | AGE  30-39 | AGE   40-49 | AGE  50-59 | AGE  60-69 | AGE  70-79 | NO. OF  ANSWERS | MIN, MAX |
|  |  |  |  |  |  |  |  |  |  |
| *Social activities (h/week)* |  |  |  |  |  |  |  |  |  |
| Time spent with: |  |  |  |  |  |  |  |  |  |
| Family members | 10.7±5.1 | 8.4±4.6 | 13.7±3.4 | 10.8±5.5 | 11.8±4.8 | 10.1±5.7 | 9.6±5.1 | 174 | 0, >15 |
| Relatives | 2.3±2.7 | 1.7±1.4 | 3.1±4.2 | 2±1.8 | 1.6±1.5 | 2.3±2 | 2.8±3.4 | 175 | 0, >15 |
| Friends | 4.9±3.8 | 7.7±4.7 | 4.7±3.9 | 3.7±2.4 | 4.5±3.2 | 3.9±2.8 | 5.2±3.9 | 176 | 0, >15 |
| Phone conversations with: |  |  |  |  |  |  |  |  |  |
| Family members | 2.5±2.4 | 1.9±1.9 | 2.5±1.8 | 2±1.7 | 2±1.2 | 2.7±2.8 | 3.7±3.6 | 177 | 0, >15 |
| Relatives | 1.4±1.7 | 0.5±0.6 | 1.1±1.2 | 1±1.1 | 1.4±1.1 | 1.8±2 | 2.4±2.8 | 176 | 0, 14 |
| Friends | 1.9±2.1 | 1.9±2.1 | 1.9±1.7 | 1.7±2.9 | 1.4±1 | 1.8±1.3 | 2.8±2.6 | 175 | 0, >15 |
| Going to restaurants/pubs/cafés with company | 2.1±2.3 | 3.2±2.8 | 2±2 | 2±2.8 | 1.9±1.6 | 2±2.1 | 1.6±1.9 | 176 | 0, 14 |
| Going to parties or social gatherings | 1.6±2.1 | 2.2±3.2 | 1.7±2.2 | 0.8±1.2 | 1.6±1.5 | 1.7±2 | 1.3±1.7 | 176 | 0, >15 |
| Going to church | 0.3±1.3 | 0.1±0.6 | 0.1±0.6 | 0.7±2.8 | 0±0 | 0.2±0.5 | 0.4±1 | 177 | 0, >15 |
| Participation in a club or union | 0.9±2 | 0.5±2 | 0.3±0.6 | 2±3.4 | 1.3±1.4 | 0.4±0.7 | 0.8±1.7 | 177 | 0, >15 |
| Total social activity | 28.6±12.2 | 28.3±10.8 | 31.2±12.3 | 26.6±11.6 | 27.5±9.7 | 26.8±11.4 | 31±16.5 | 169 | 4, 78 |
| *Cognitive activities (h/week)* |  |  |  |  |  |  |  |  |  |
| Driving a car | 4.2±3.7 | 3.3±3.4 | 4.7±4.3 | 5.2±4.3 | 4.4±3.2 | 3.9±3.4 | 3.8±3.3 | 177 | 0, >15 |
| Play computer games | 1.4±3.3 | 3.4±5.2 | 2.3±4.2 | 0.8±1.5 | 0.4±1.9 | 0.5±1.5 | 1.1±3.1 | 178 | 0, >15 |
| Using the computer (other than games) | 5.9±5.1 | 8.2±5.3 | 5.5±5.4 | 7±6 | 7.9±5.1 | 3.8±3.3 | 3.8±3.8 | 176 | 0, >15 |
| Cooking | 5.8±3.4 | 5.6±3 | 5.9±2.9 | 5.5±2.7 | 6.9±3.4 | 5.1±3.9 | 5.8±4.1 | 176 | 0, >15 |
| Reading non-fiction (sv. faktalitteratur) | 1.8±2.5 | 2.8±3.7 | 1.6±2.4 | 2.1±2.9 | 1.6±1.4 | 1.3±1.6 | 1.2±2.1 | 178 | 0, >15 |
| Reading novels | 2.6±3.2 | 2±2.6 | 2.8±3.5 | 2.9±3 | 2.2±2.6 | 2.7±3.3 | 3.2±3.8 | 178 | 0, >15 |
| Reading papers (sv. morgontidningen) | 2.1±2.2 | 0.8±1 | 1.4±1.6 | 1.4±1.7 | 2.4±2.1 | 3.1±2.3 | 3.2±2.7 | 178 | 0, 10 |
| Reading newspapers or periodicals | 1.2±1.6 | 0.6±1 | 0.9±1.3 | 0.7±0.9 | 1.3±1.8 | 1.6±1.5 | 2±2.1 | 178 | 0, 8 |
| Going to lectures or to other education | 0.7±2.6 | 3±5.2 | 0.5±2.7 | 0.2±0.5 | 0.1±0.4 | 0.1±0.3 | 0.4±1 | 178 | 0, >15 |
| Studying at home | 0.4±1.8 | 1.6±3.4 | 0.1±0.4 | 0.6±2.4 | 0.2±0.6 | 0.1±0.3 | 0.2±0.8 | 177 | 0, >15 |
| Playing cards | 0.8±1.5 | 1.8±2.9 | 0.6±0.9 | 0.7±0.9 | 0.7±1.1 | 0.4±0.9 | 0.5±0.9 | 178 | 0, >15 |
| Playing board games | 0.4±1.4 | 0.9±1.1 | 1.1±2.8 | 0.4±0.9 | 0.2±0.5 | 0±0.2 | 0±0.2 | 178 | 0, >15 |
| Playing a musical instrument | 0.5±1.9 | 2.1±4.2 | 0.2±0.6 | 0.3±0.9 | 0.1±0.3 | 0.3±1 | 0.1±0.4 | 177 | 0, >15 |
| Going to museums or art exhibitions | 0.4±0.8 | 0.2±0.8 | 0.3±0.6 | 0.1±0.4 | 0.3±0.5 | 0.6±1 | 0.7±1 | 178 | 0, 4 |
| Crossword puzzles | 1±1.8 | 0.3±0.8 | 0.9±1.7 | 0.7±1.3 | 1±1.6 | 1.1±1.5 | 2.1±2.5 | 178 | 0, 10 |
| Riddles and mind puzzles (e.g. Sudoku) | 0.8±1.7 | 1.4±2.9 | 0.6±0.7 | 0.9±1.7 | 0.8±1.7 | 0.5±0.9 | 0.6±1.4 | 178 | 0, >15 |
| Writing texts | 3±3.5 | 3±4.5 | 4±4 | 3.1±2.8 | 3.7±3.8 | 2±2.7 | 2.5±2.6 | 178 | 0, >15 |
| Calculating | 2.5±3 | 3.3±4.6 | 3.3±3.7 | 2.5±2.8 | 2.6±2.2 | 1.9±1.6 | 1.7±1.9 | 178 | 0, >15 |
| Total cognitive activity | 35.5±17.5 | 44.2±18.7 | 36±17.5 | 35.5±16.4 | 36.3±17.3 | 29±14.5 | 32.7±18 | 172 | 4, 87 |
| *Physical activities (h/week)* |  |  |  |  |  |  |  |  |  |
| Gardening | 3±3.7 | 0.6±0.8 | 2.6±2.9 | 3.2±4.1 | 2.9±3.3 | 3.9±3.4 | 4.7±4.9 | 178 | 0, >15 |
| Cleaning | 2.7±2.1 | 2.4±1.7 | 3.4±3 | 3±2.7 | 2.1±1.1 | 2.6±1.5 | 2.5±1.9 | 178 | 0, >15 |
| Dancing | 0.5±1.1 | 0.7±1 | 0.6±0.9 | 0±0.2 | 0.4±1.3 | 0.5±1.3 | 0.5±1.2 | 178 | 0, 7 |
| Walking | 5.7±3.7 | 5.1±2.8 | 6.6±3.5 | 4.9±3.6 | 5.6±3.5 | 6.2±4 | 5.7±4.6 | 178 | 0, >15 |
| Cycling | 3.3±3.4 | 2.6±1.9 | 3.5±2.9 | 3.4±2.9 | 3.5±3.4 | 4.1±4.3 | 2.9±4.2 | 177 | 0, >15 |
| Bodybuilding (sv. styrketräning) | 1.4±2.3 | 2.6±3.2 | 1.5±2.3 | 1.4±2.3 | 1.6±2.1 | 0.6±1.3 | 0.6±1.8 | 178 | 0, 12 |
| Gymnastics/aerobics (sv. gympa) | 0.5±1 | 0.3±0.8 | 0.3±0.6 | 0.4±0.9 | 0.6±1.2 | 0.6±1.2 | 0.5±1.4 | 177 | 0, 7 |
| Jogging | 1.1±1.8 | 2±1.6 | 1.4±2.2 | 1.2±1.5 | 1.2±1.6 | 0.6±1.2 | 0.6±2 | 178 | 0, 10 |
| Roller skiing (sv. åka rullskidor) | 0.1±0.8 | 0.4±1.9 | 0.1±0.5 | 0±0 | 0±0.2 | 0.1±0.5 | 0±0 | 178 | 0, 10 |
| Fishing | 0.6±1.6 | 0.3±0.5 | 0.3±0.7 | 0.4±1.2 | 0.9±2.8 | 0.6±1.3 | 0.9±1.9 | 178 | 0, >15 |
| Sailing | 0.1±0.5 | 0.4±1.3 | 0±0 | 0±0 | 0±0 | 0±0 | 0±0 | 177 | 0, 5 |
| Hunting | 0.3±1.5 | 0.1±0.6 | 0.4±1.2 | 0±0.2 | 1.1±3.3 | 0.1±0.3 | 0±0 | 178 | 0, >15 |
| Collecting mushrooms in forest | 0.8±1.7 | 0.4±0.6 | 1±1.6 | 0.7±1.5 | 0.7±1.6 | 1.1±1.5 | 1.1±2.5 | 177 | 0, 12 |
| Doing sports (i.e. tennis, golf, bowling) | 1.2±2.9 | 2.2±3.7 | 0.6±0.9 | 1.3±2.8 | 1.1±2 | 1±3.1 | 1.2±3.8 | 178 | 0, >15 |
| Flexibility and coordination classes | 0.7±1.8 | 1.3±2.9 | 0.7±1.5 | 0.8±1.8 | 1±2 | 0.4±1 | 0.3±0.8 | 178 | 0, >15 |
| Total physical activity | 21.9±12.5 | 21±10 | 22.9±9.9 | 20.8±14.4 | 22.7±11.3 | 22.5±12.4 | 21.7±16.1 | 174 | 3, 79 |

*Answers “>15” are treated as 15 in calculations of means and standard deviations. **Highest observed mean values are marked in bold.

**S.5. Normality of cognitive test scores**

Figure 3 reveals marked differences between distributions of responses across cognitive tests. Among episodic memory tasks, the scores for number-word recall were the most skewed (skewness = 1.32, kurtosis = 5.08, see Figure 3A). We did not find evidence for non-normality of the scores for the object-recall test (Shapiro-Wilk test, W=0.99, n=180, p-value = 0.16, skewness = 0.05 and kurtosis = 2.5), whereas slight skewness was observed for word recall (skewness = 0.44, kurtosis = 2.27). The scores for the letter-updating and number-updating tasks were skewed to the left (skewness = -1 and -1.24, respectively, kurtosis = 3.83- 3.87). The number-updating task, for example, had a high proportion of high scores from younger individuals (see Figure 3B), whereas most of the low scores in this task came from the older subjects. Spatial updating was the most symmetrical among the working memory tasks (skewness = 0.33, kurtosis = 2.77). Moderate skewness was observed in all perceptual speed tasks (skewness = 0.49-0.91, kurtosis = 2.69-5.3). Additionally, some subjects performed either very poorly or outstanding in the figure-comparison and number-comparison tasks.

The scores for the semantic knowledge task were moderately skewed to the left (skewness = -0.83, kurtosis = 3.05), whereas the scores for the implicit learning task had a more symmetric distribution (skewness = 0.42, kurtosis = 3.87). Finally, some subjects had performed poorly in the finger-tapping task for the left hand (skewness = -0.22, kurtosis = 4.51), although the distribution of the scores for tapping with the right hand was approximately symmetric (skewness 0.01, respectively, kurtosis = 4).

**S.6. Gray matter volumes**

Distributions of adjusted gray-matter volumes are presented in Supplementary Figure 1A. Distributions of cingulate cortex and hippocampus volumes showed negative skewness (Shapiro-Wilks test; cingulate cortex: W = 0.97; n = 177; *p* < 0.001; skewness = -0.46, kurtosis = 5.03; hippocampus: W = 0.98; n = 177; *p* = 0.03; skewness = -0.45; kurtosis = 3.12), whereas no evidence of non-normality was found in frontal cortex (W = 0.99; n = 177; p = 0.71), caudate (W = 0.99; n = 177; p = 0.16), putamen (W = 0.99; n = 177; p = 0.23), and nucleus accumbens (W = 0.99; n = 177; p = 0.99). Age-stratified box-plot analysis (Figure 1B) showed no outliers outside biologically plausible ranges. Average gray matter volumes (cm^3^) were 179.85±13.05 in frontal cortex, 20.50±2.18 in cingulate cortex, 9.57±1.03 in putamen, 8.45±0.66 in hippocampus, 7.21±0.76 in caudate, and 1.12±0.17 in nucleus accumbens.


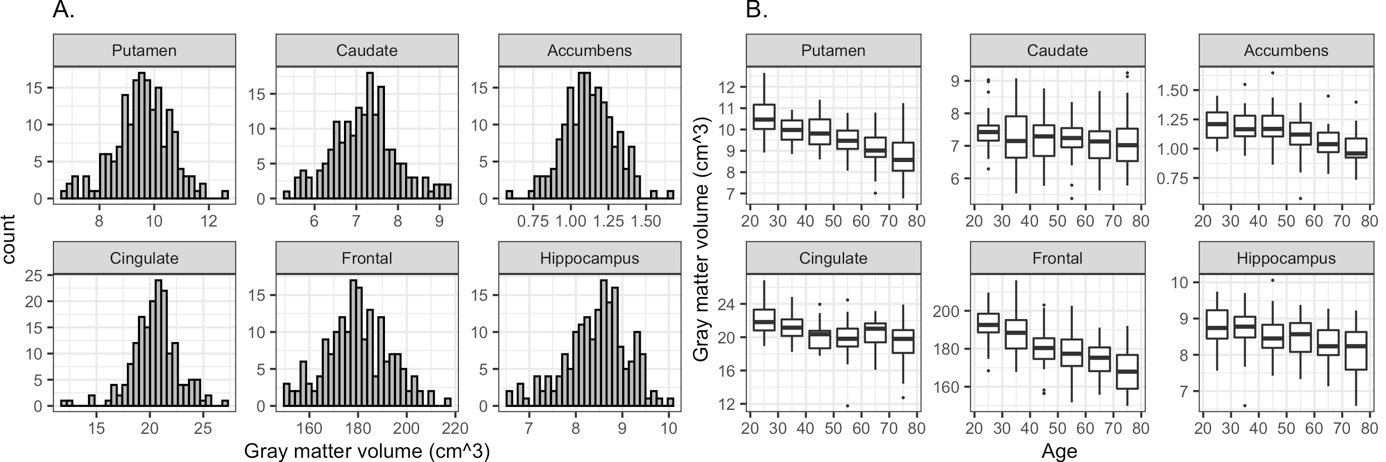


*Supplementary Figure 1.* A) Histograms portraying distributions of adjusted regional gray matter volumes (cm^3^) for the putamen, caudate nucleus, nucleus accumbens, cingulate cortex, frontal lobe, and hippocampus. B) Adjusted gray matter volumes across age groups.

**References**

Avelar-Pereira, B., Bäckman, L., Wåhlin, A., Nyberg, L., & Salami, A. (2017). Age-Related Differences in Dynamic Interactions Among Default Mode, Frontoparietal Control, and Dorsal Attention Networks during Resting-State and Interference Resolution. *Frontiers in Aging Neuroscience*, *9*. https://doi.org/10.3389/fnagi.2017.00152

Björnfot, C. (2018). *Multiband functional magnetic resonance imaging (fMRI) for functional connectivity assessments.* [Umeå University]. https://www.diva-portal.org/smash/record.jsf?pid=diva2:1228907

Cole, M. W., Bassett, D. S., Power, J. D., Braver, T. S., & Petersen, S. E. (2014). Intrinsic and Task-Evoked Network Architectures of the Human Brain. *Neuron*, *83*(1), 238–251. https://doi.org/10.1016/j.neuron.2014.05.014

Finn, E. S., Scheinost, D., Finn, D. M., Shen, X., Papademetris, X., & Constable, R. T. (2017). Can brain state be manipulated to emphasize individual differences in functional connectivity? *NeuroImage*, *160*, 140–151. https://doi.org/10.1016/j.neuroimage.2017.03.064

Geerligs, L., Rubinov, M., Cam-CAN, & Henson, R. N. (2015). State and Trait Components of Functional Connectivity: Individual Differences Vary with Mental State. *Journal of Neuroscience*, *35*(41), 13949–13961. https://doi.org/10.1523/JNEUROSCI.1324-15.2015

Greene, A. S., Gao, S., Scheinost, D., & Constable, R. T. (2018). Task-induced brain state manipulation improves prediction of individual traits. *Nature Communications*, *9*(1), 2807. https://doi.org/10.1038/s41467-018-04920-3

Hasson, U., Malach, R., & Heeger, D. J. (2010). Reliability of cortical activity during natural stimulation. *Trends in Cognitive Sciences*, *14*(1), 40–48. https://doi.org/10.1016/j.tics.2009.10.011

McArdle, J. J., & Nesselroade, J. R. (1994). Using multivariate data to structure developmental change. In *Life-span developmental psychology: Methodological contributions,* (pp. 223–267). Psychology Press.

Nevalainen, N., Riklund, K., Andersson, M., Axelsson, J., Ögren, M., Lövdén, M., Lindenberger, U., Bäckman, L., & Nyberg, L. (2015). COBRA: A prospective multimodal imaging study of dopamine, brain structure and function, and cognition. *Brain Research*, *1612*, 83–103. https://doi.org/10.1016/j.brainres.2014.09.010

Nilsson, L.-G., Adolfsson, R., Bäckman, L., Frias, C. M. de, Molander, B., & Nyberg, L. (2004). Betula: A Prospective Cohort Study on Memory, Health and Aging. *Aging, Neuropsychology, and Cognition*, *11*(2–3), 134–148. https://doi.org/10.1080/13825580490511026

Rosseel, Y. (2012). lavaan: An R package for structural equation modeling and more Version 0.5-12 (BETA). *Journal of Statistical Software*, 37.

Salami, A., Garrett, D. D., Wåhlin, A., Rieckmann, A., Papenberg, G., Karalija, N., Jonasson, L., Andersson, M., Axelsson, J., Johansson, J., Riklund, K., Lövdén, M., Lindenberger, U., Bäckman, L., & Nyberg, L. (2019). Dopamine D2/3 binding potential modulates neural signatures of working memory in a load-dependent fashion. *Journal of Neuroscience*, *39*(3), 537–547. https://doi.org/10.1523/JNEUROSCI.1493-18.2018

Salami, A., Rieckmann, A., Karalija, N., Avelar-Pereira, B., Andersson, M., Wåhlin, A., Papenberg, G., Garrett, D. D., Riklund, K., Lövdén, M., Lindenberger, U., Bäckman, L., & Nyberg, L. (2018). Neurocognitive profiles of older adults with working-memory dysfunction. *Cerebral Cortex*, *28*(7), 2525–2539. https://doi.org/10.1093/cercor/bhy062

Vanderwal, T., Eilbott, J., Finn, E. S., Craddock, R. C., Turnbull, A., & Castellanos, F. X. (2017). Individual differences in functional connectivity during naturalistic viewing conditions. *NeuroImage*, *157*, 521–530. https://doi.org/10.1016/j.neuroimage.2017.06.027
